# A high-fidelity microfluidic platform reveals retrograde propagation as the main mechanism of α-Synuclein spread in human neurons

**DOI:** 10.1038/s41531-025-00936-x

**Published:** 2025-04-20

**Authors:** Rozan Vroman, Lorenzo de Lichtervelde, Karamjit Singh Dolt, Graham Robertson, Marco Kriek, Michela Barbato, Justyna Cholewa-Waclaw, Tilo Kunath, Patrick Downey, Michele Zagnoni

**Affiliations:** 1 Center for Microsystems and Photonics, Department of Electronic and Electrical Engineering, University of Strathclyde, Glasgow, UK; 2 UCB Biopharma, Chemin du Foriest, 1420 Braine-l’Alleud, Belgium; 3 Centre for Regenerative Medicine, Institute for Regeneration and Repair, The University of Edinburgh, Edinburgh, UK; 4 UCB Biopharma UK, Slough, UK; 5 Institute for Stem Cell Research, School of Biological Sciences, The University of Edinburgh, Edinburgh, UK

**Keywords:** Neural stem cells, Diseases of the nervous system

## Abstract

α-Synuclein (αSyn) is a major component of Lewy bodies and Lewy neurites, which are a pathological hallmark of Parkinson’s disease (PD). Pathologically aggregated forms of αSyn can spread along neurites and induce the misfolding of normal αSyn. To elucidate how αSyn pathology propagates between brain areas, we developed a novel in vitro microfluidic platform to study the intracellular transport of preformed fibrils and the induction and spread of αSyn aggregates. Patient-derived midbrain dopaminergic (mDA) neurons were cultured in microfluidic devices designed to maintain unidirectional axonal connections between fluidically isolated mDA neuronal cultures for over 3 months. Using αSyn preformed fibrils to induce Lewy-like pathology, we found that anterograde spread of αSyn fibrils was slow and occurred at low levels, while retrograde spread was significantly more efficient. This is in line with observations in animal models and shows that the platform provides an innovative new tool for studying PD in vitro.

## Introduction

α-Synuclein (αSyn) is localizes to a subset of synapses and nuclei in the central nervous system^1^ and has been intimately linked to Parkinson’s disease (PD) since 1997, when the first familial cases of PD were shown to harbour a single amino acid change in this protein^2^. αSyn is intrinsically disordered in solution, but upon agitation, can form elongated self-aggregating amyloid fibrils that resemble the fibrils found in Lewy bodies (LBs)^3–5^. As well as being aggregated, the vast majority of αSyn in LBs was found to be phosphorylated especially at position serine-129^6^. The pioneering work of Heiko Braak first showed that αSyn pathology spreads throughout the brain of Parkinson’s patients in a stereotypical caudo-rostral pattern^7^. Recent studies have confirmed that this spread pattern is seen in 80–90% of typical PD cases^8,9^. A potential mechanism of how αSyn may spread through the brain has come from in vitro studies which show that aggregated αSyn can template monomeric αSyn to mis-fold and form aggregates^10–14^. Templated aggregation has been shown to occur in cultured cells^15^, rodent primary neurons^16^, human mDA neurons^17^ and organotypic brain organoids^18^, as well as multiple in vivo models where αSyn pathology spreads widely throughout the brain^19–23^.

Cellular studies with mouse cortical neurons^24,25^ and human induced pluripotent stem cell (hiPSC)-derived cortical neurons^26^ suggest that the propagation of Lewy-like pathology occurred by both anterograde and retrograde intracellular spread as well as cell-to-cell transfer, while recent in vivo results reported that αSyn pathology is primarily retrogradely propagated^27–29^. Studies in human tissue also propose retrograde spread as the main mechanism^30,31^. This discrepancy between in vivo and in vitro studies has not been previously addressed.

As PD most significantly affects mDA neurons, the use of hiPSC-derived mDA cells has the potential to provide a physiologically relevant human model to study the spread of pathological synuclein in vitro. Combining this human cell model with microfluidic techniques, designed to ensure unidirectional axonal growth^32^ and functional synaptic connections^33,34^, has the potential to provide a powerful technology to study the mechanisms of pathological αSyn propagation.

In the context of PD, microfluidic technologies^35,36^ have been previously used to study the pathological spread of αSyn^24–26^, especially for the ability to control unidirectional neurite growth in vitro. However, there are inherent difficulties in obtaining and maintaining such cellular topological conditions during increasingly long culture times. This is especially relevant when using hiPSC-derived neurons, as these cells typically require extensive culture periods (up to 3 months) to acquire a mature and functional neuronal phenotype^37,38^ and to develop significant αSyn pathology^17^. Previously reported microfluidic layouts (e.g., using tapered microchannels or axonal edge-guiding features) could achieve unidirectional axonal growth for a limited time (i.e., under a month^26,39–43^) and showed a 3–5% failure in achieving the goal^40^. Therefore, there remain considerable challenges to create high-fidelity assays when using long-term cultured hiPSC neurons to study anterograde and retrograde propagation of prion-like pathology in vitro.

This work had a dual aim: to create a robust microfluidic platform for unidirectional neurite growth using hiPSC mDA neurons and to develop associated, bespoke protocols to study the spread of pathological αSyn. Our objectives were to distinguish between intracellular transport of preformed fibrils (PFFs), the transport of pathological aggregates and the cell-to-cell transfer of αSyn pathology. To this end, we first compared the efficiency of a variety of existing and novel microfluidic layouts and methodologies to maximise the chances of achieving unidirectional connections between two compartmentalised neuronal cultures over several months in culture. For this, we used the α-synuclein triplication 18 (AST18) hiPSC line that significantly over-expresses αSyn when differentiated into mDA neurons^44^. Subsequently, we developed an in vitro model that mimics the pathological spread of αSyn, using AST18 hiPSC-derived mDA cells and αSyn PFFs to initiate the seeding of Lewy-like pathology. Using this model, we developed new assays to assess different possible propagation mechanisms of synuclein pathology. We showed that αSyn pathology is more efficiently propagated in the retrograde direction than in the anterograde direction. This is likely due to αSyn PFFs being transported retrogradely from the axon towards the soma via intracellular mechanisms. Instances were found showing that αSyn pathology was transferred from cell to cell only to a small extent in our experimental conditions. These results are in line with recently reported findings in in vivo mouse models and demonstrate that the developed platform provides a powerful in vitro tool for studying PD mechanisms and for testing treatments aimed at reducing the spread of αSyn pathology.

## Results

PD is characterised by the dysfunction and loss of substantia nigra neurons within the midbrain. We therefore made use of AST18 hiPSCs and differentiated them into mDA neurons^44^. This cell line was derived from a patient with a triplication of *SNCA* and expresses twice the amount of αSyn protein when compared to control neurons, making it more susceptible to developing αSyn pathology^44^. AST18 hiPSCs were differentiated into mDA progenitor cells and neurons using a modified midbrain floor plate method (Supplementary Fig. 1A)^45–47^. The midbrain identity of the cells was checked by immunostaining for the transcription factors LMX1A and FOXA2, markers of midbrain floor plate cells in vivo^48,49^. At day 16 of differentiation the majority of cells had converted to LMX1A:FOXA2 double-positive mDA neural progenitor cells (Supplementary Fig. 1B) and lineage-committed mDA progenitor cells were cryopreserved using an established protocol^50^. Progenitor cells produced using our method can rescue the 6-hydroxydopamine lesion rat model of Parkinson’s upon transplantation^51^, and upon maturation in culture they exhibit spontaneous electrophysiological activity and dopamine release^52^. All batches of frozen mDA progenitor cells were test-thawed in bulk culture and differentiation was resumed beyond day 40 before assaying for expression of the dopaminergic marker tyrosine hydroxylase (TH) (Supplementary Fig. 1C). Furthermore, at day 103 of differentiation we observed significant overlap of βIII-tubulin (TuJ1) and TH expression in our first-generation bidirectional devices (Supplementary Fig. 1D). The quality-controlled mDA progenitor cells were plated in microfluidic devices and allowed to mature into mDA neurons for up to 21 weeks.

### Validation of unidirectional axonal growth in microfluidics

**Fig. 1:**
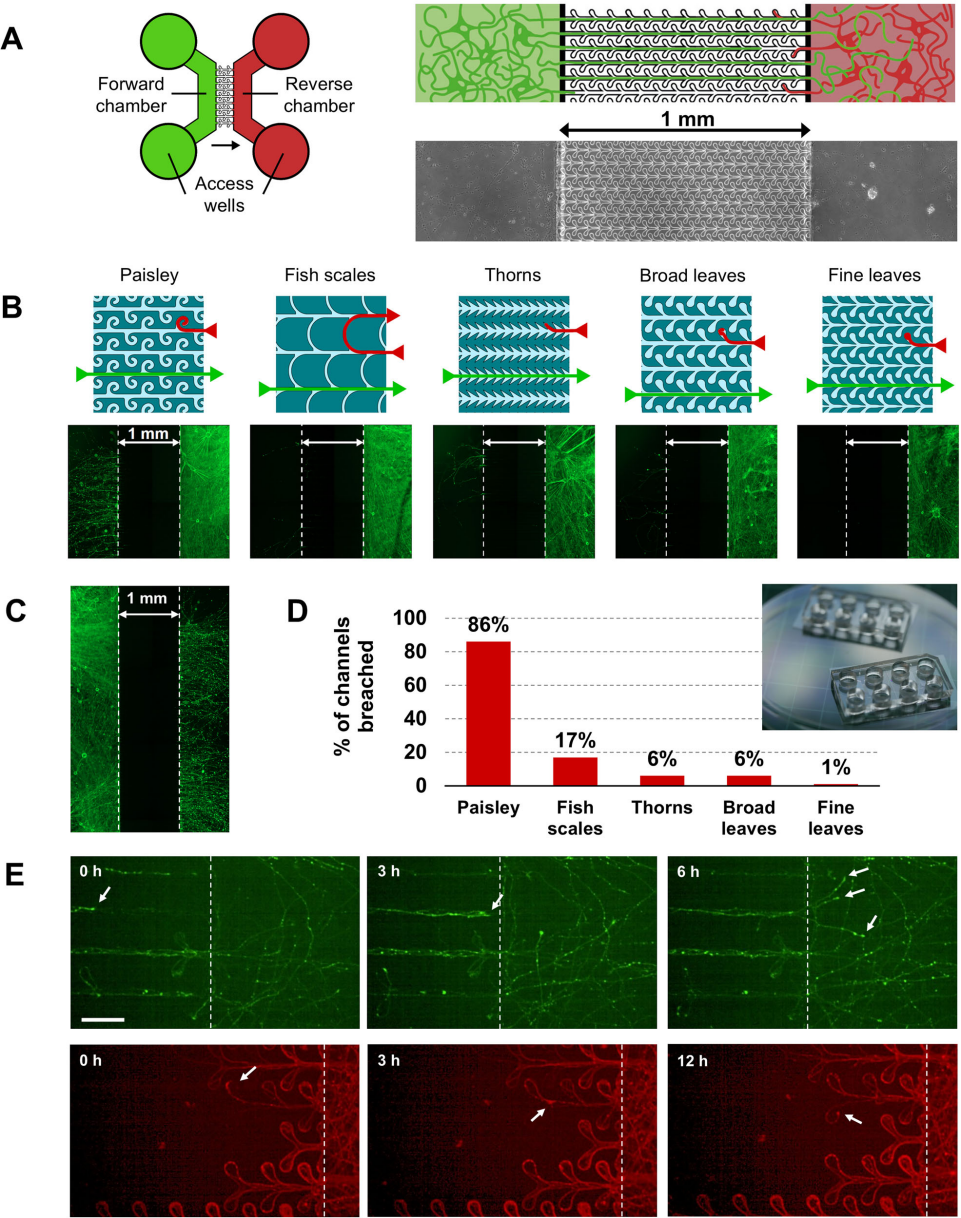
The “fine leaves” pattern was most successful at preventing reverse-originating axons to reach the forward chamber. **A** Schematic of the device. Left: The design of the microfluidics device consisting of two chambers that are connected by the patterned microchannels such that axons can only reach the other chamber from left to right. The circles are the wells that give access to the chambers and are used for plating cells, refreshing the medium and seeding PFFs. The green side is the forward side and the red the reverse side. The arrow indicates the direction in which neurites are allowed to grow unhindered. Right: Close-up of the schematic to show the microchannels and underneath an image of the actual device. **B** Schematics of the 5 microchannel patterns and underneath fluorescence microscopy image of devices stained with βIII-tubulin showing the effectiveness of the unidirectional design. mDA cells were plated in the Reverse chamber only. The ‘fine leaves’ design was most effective in preventing axons from crossing to the Forward chamber. Note that the neurites within microchannels need a longer exposure-time and are therefore not visible in this example. **C** Axons from neurons cultured in the forward chamber were not hindered in reaching the reverse chamber, here shown for the ‘fine leaves’ design. **D** Graph showing the varying extent to which the microchannels of different design were breached. **E** Mouse hippocampal neurons expressing EGFP were cultured in the forward chamber while neurons expressing m-cherry were simultaneously cultured in the reverse chamber (or vice versa). Images are shown from a time-lapse experiment. Top: temporal progression of a microfluidic culture showing forward-originating axons reaching the reverse chamber. Arrows indicate example growth cones. Bottom: temporal progression showing axons originating in the reverse chamber remaining trapped in the ‘dead-end’ leaves of the microfluidic pattern or reversing growth direction and returning towards the originating chamber. The scale bar is 50 µm.

To assess both the directional propagation of pSyn pathology from one culture chamber to another (Fig. 1A), as well as to investigate anterograde and retrograde axonal transport mechanisms, we designed and tested new microfluidic layouts and compared their performance with previously reported ones^26,39–43^ purported to achieve unidirectional axonal growth. All our designs used neurite edge-guiding behaviour to redirect and entrap the axonal processes (Fig. 1B and Supplementary Fig. 2). To test the efficiency of the different microchannel patterns, we used a two-chamber configuration (Fig. 1A) with the chambers spaced 1 mm apart (length of microchannel barrier). This longer than usual microchannel length was used to account for the extended culture times and to ensure that only axons could reach the opposite chamber^53^ (Supplementary Fig. 3). Initial experiments were conducted by plating mDA progenitor cells in either the forward or reverse growth chambers with a culture duration of 10 weeks. Neurons were then immuno-stained for βIII-tubulin expression and imaged to assess the extent of forward and reverse axonal growth for each microchannel pattern. When cells were plated in the forward chamber, axons were able to reach the reverse chamber for all patterns tested (Fig. 1C), while in the opposite condition, reverse axonal growth varied according to the different geometrical patterns (Fig. 1B, D). The best results in limiting reverse axonal projections were obtained for a novel microchannel pattern, termed ‘fine leaves’, which achieved 99% unidirectional axonal growth (Fig. 1D).

Unidirectional axonal growth was further assessed in the ‘fine leaves’ pattern devices after ~13 and ~21 weeks of culture (Supplementary Fig. 4). At Day 114 of differentiation (13.1-week microfluidic assay, 4 devices), no axons originating in the reverse chamber had reached the forward chamber. At Day 172 (21.4-week microfluidic assay, 4 devices) in only 1 instance, four breaches of axon growing into the reverse chamber were observed (1.25% failure). These results suggest that neuronal cultures in the ‘fine leaves’ pattern devices remained unidirectionally connected for at least 13 weeks, with a very low probability (≤1%) of reverse neurite growth if studied within a timeframe of 20 weeks.

Further, to validate the performance of the ‘fine leaves’ microchannel designs to produce unidirectional axonal growth, neurons expressing different fluorophores were cultured simultaneously in both chambers. In this instance, we used primary hippocampal mouse neurons, where one of the cultures was infected with a viral vector to express EGFP-tagged or mCherry tagged α-synuclein (the same infection procedure was unsuccessful with hiPSCs). Live cell imaging was performed using confocal microscopy over a 24-hour period. When observing neurons plated in the forward chamber, axons were seen reaching the reverse chamber with ease (Fig. 1E top; Supplementary video 1A), whereas the axons of neurons plated in the reverse chamber were found to remain trapped within the leaf ‘dead-end’ feature of the microchannels or indeed doubled back on themselves (Fig. 1E bottom; Supplementary video 1B), reversing growth direction back to the originating chamber. Additionally, when performing live-cell imaging of hiPSC mDA neurons using brightfield, a similar behaviour was observed (Supplementary video 2).

As a result, the ‘fine leaves’ microchannel pattern was used in all subsequent experiments with a typical duration of microfluidic assays of around 13 weeks and never exceeding 20 weeks. Lastly, to prevent PFFs from being accidentally transported by flow or diffusion via extracellular mechanisms across the microchannels, fluidic isolation between chambers was maintained by ensuring that the liquid levels in the wells of the non-seeded chamber was always higher than in those connected to the seeded chamber for the entire duration of the assay, thus establishing a hydrostatic pressure gradient. The efficiency of fluidic isolation was validated by fluorescence microscopy using calcein, a molecule with a MW much smaller than PFFs (Supplementary Fig. 5). This ensured seeding was restricted to only one of the chambers.

### Induction and characterization of phosphorylated αSyn pathology in human mDA neurons

To induce the formation of Lewy-like pathology in mature human mDA neurons, we used recombinant αSyn preformed fibrils (PFFs) as previously described^54^. When PFFs enter the neurons, they template the normally soluble αSyn protein causing it to misfold and become phosphorylated at serine-129 (pSyn). This results in the formation of de novo insoluble aggregates which accumulate within neurites and cell bodies. Immunofluorescence of αSyn phosphorylated at serine-129 (pSer-129-αSyn), a disease-specific post-translational modification, was used as a measure of molecular pathology, quantifying both the total area of pathology and the number of pSyn fragments induced by PFFs (Supplementary Fig. 6). Only pSer-129-αSyn staining that co-localised with βIII-tubulin neuronal staining was considered for quantitative analysis. These values were tested against control experiments to which no PFFs were added (unseeded cultures).

Significant pSer-129-αSyn levels were obtained only at approximately 30 days post PFF seeding with respect to unseeded controls. Small and sporadic pSyn fragments appeared, which progressively increased in length and number over time, with several clusters of sequential pathology located within neurites. In some instances, larger pSer-129-αSyn positive structures were also observed (Supplementary Fig. 7). To assess whether these areas could represent an early sign of Lewy-body formation, we tested these structures for the expression of p62/SQSTM1 and microtubule-associated protein-2 (MAP2). The ubiquitin-binding protein p62 is found in Lewy bodies^55^. In addition to staining dendrites, MAP2 has been reported to co-localise with pSyn and ubiquitin in Lewy bodies found in the substantia nigra of PD patients^56^. Structures showing very strong labelling for pSer-129-αSyn often co-localized with both p62 and MAP2 (Supplementary Fig. 7), markers which are also used to detect pathological Lewy bodies. In the triple-positive Lewy body-like structures, the pSer-129-αSyn area most often encompassed a p62/MAP2 core region (Supplementary Fig. 7). Furthermore, pSer-129-αSyn-positive staining showed co-localisation with LC3B and HSP70 (Supplementary Fig. 8) and was detergent-resistant (1% Triton-X; Supplementary Fig. 9). Occasional non-specific or background pSer-129 positive staining was also observed in some dendrites and cell somas (Supplementary Fig. 10), this was characterised by low intensity and ragged or soft edges.

### Labelled PFFs are transported in the retrograde but not anterograde direction

**Fig. 2:**
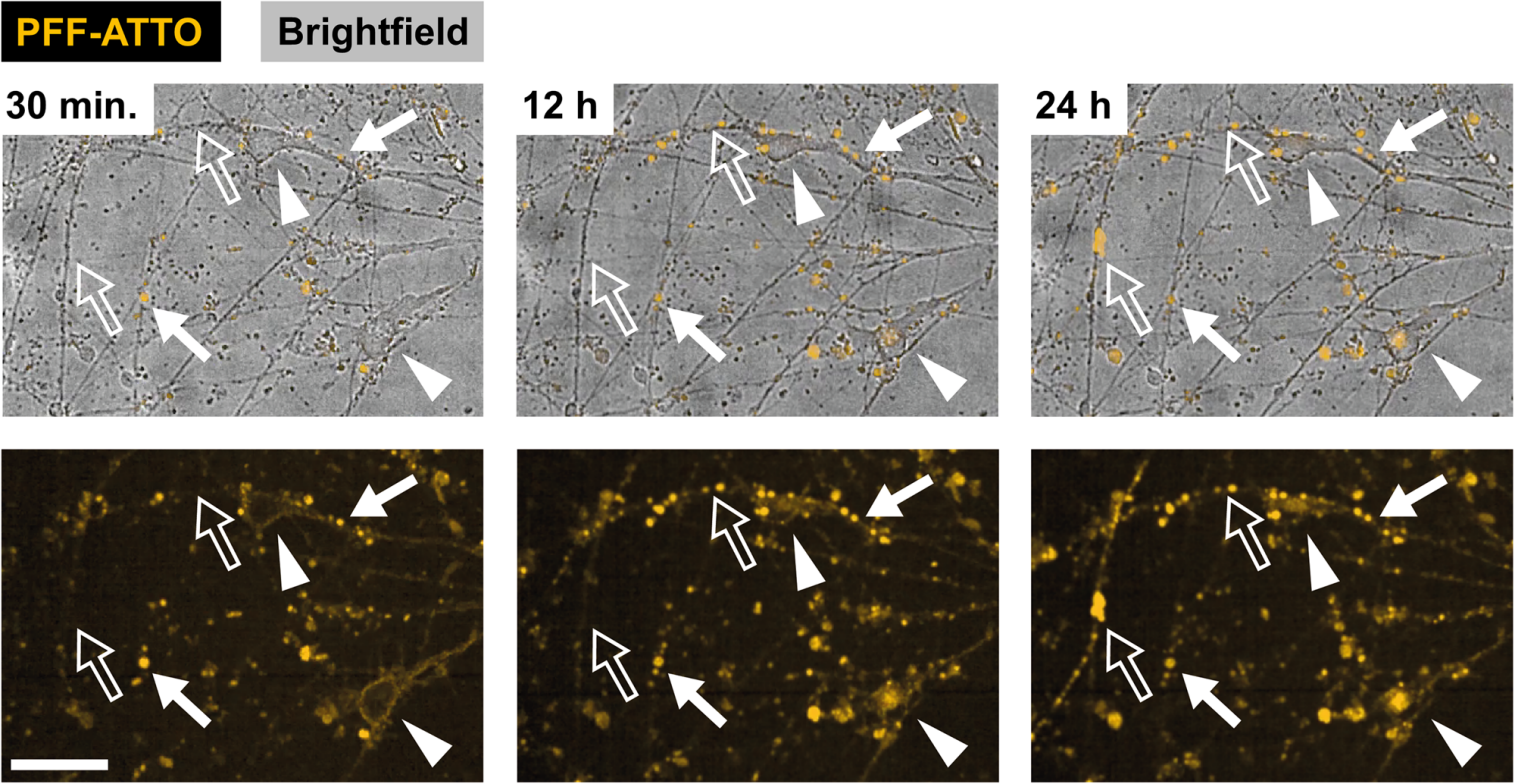
PFFs labelled with an ATTO dye (yellow) were added to one of the cultured chambers and images were taken every 15 min for a total of 24 h. Shortly after adding the PFF-ATTO puncta were visible on neurites. The filled arrows show puncta that were present early on and were stationary over time. The open arrows indicate locations on neurites where puncta appeared later in time. The arrowheads indicate cell bodies that initially only show labelling on the outside, but then gradually fill up over time. The experiment was repeated in 4 devices. The scale bar indicates 50 µm.

AST18 hiPSC-derived mDA neurons were plated in both chambers of microfluidic devices. As an additional measure to limit reverse-originating axons reaching the forward chamber, twice as many cells were plated in the forward chamber than in the reverse chamber. PFFs labelled with an ATTO-568 dye were seeded in either the forward or reverse chamber to assess transport in respectively the anterograde and retrograde direction. Confocal time-lapse imaging was employed to monitor the presence of PFFs in both chambers and within the microchannels over a period of 24 h. Immediately after addition, the labelled PFFs could be observed on the outside of the cell somas and puncta were visible on neurites (Fig. 2, left). Over time the number of stationary puncta on neurites increased and some somas gradually filled with labelled PFFs (Fig. 2; Supplementary video 2). Whereas most puncta in neurites remained stationary over time, some small dots were observed to travel along neurites.

Initially, to assess transport through the microchannels, images were taken every 15 min for a 24 h period and several randomly selected fields of view covering a section of the microchannels were analysed by making kymographs of each microchannel, taking a maximum projection over the width of the channel (excluding the area of leaves patterns). When PFFs were seeded in the reverse chamber, small puncta started to appear after about 6 h in 5 of the 26 channels analysed (2 devices; Supplementary Fig. 11), suggesting that a small number of PFFs were transported in the retrograde direction. When PFFs were seeded in the forward chamber, however, none of the 27 channels (2 devices) analysed showed transient puncta, suggesting that anterograde transport was either absent or very rare.

**Fig. 3:**
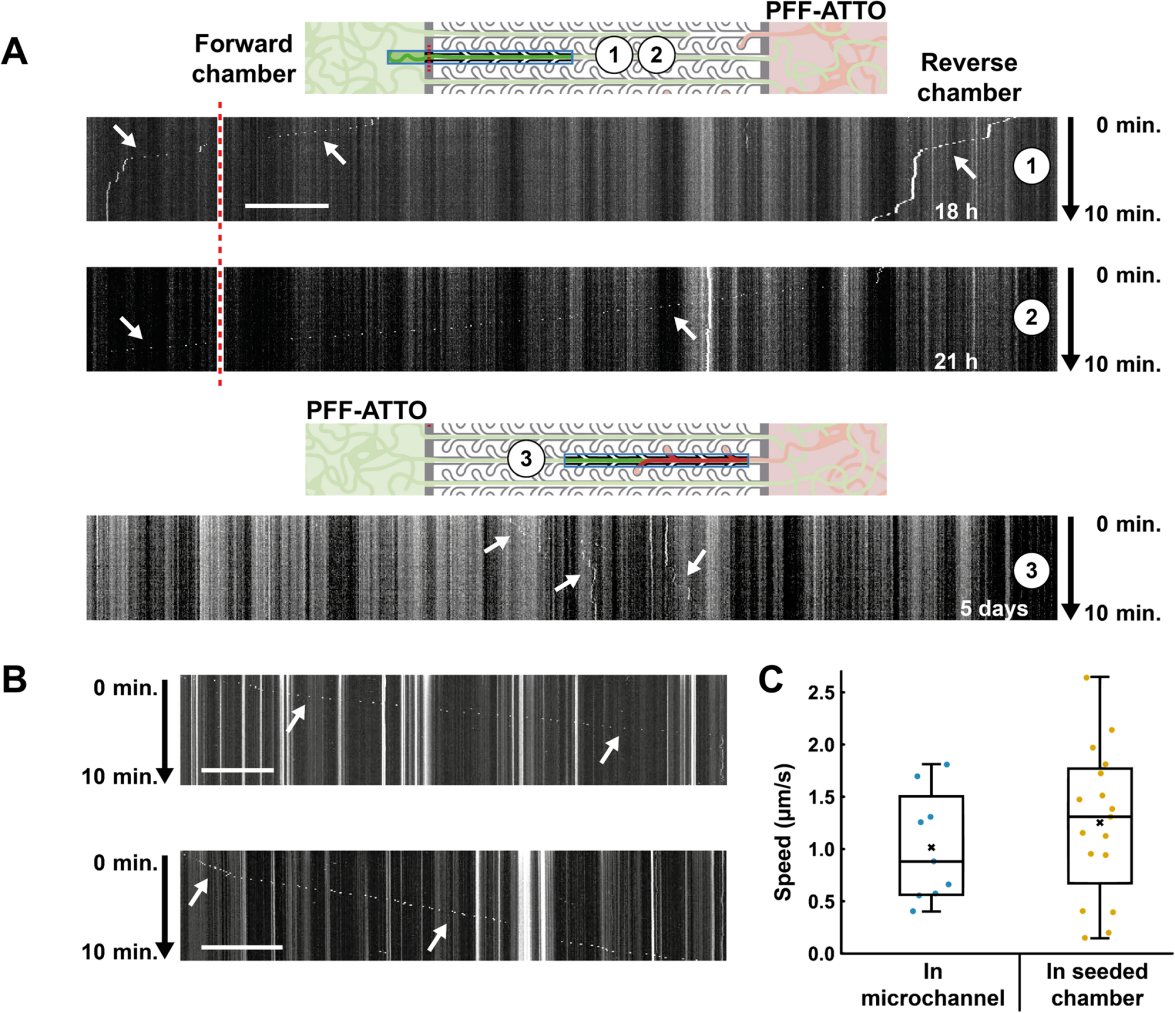
PFF-ATTO is transported retrogradely but not anterogradely. **A** (top) PFF-ATTO was seeded in the reverse chamber. Kymographs were produced from a section of a microchannel including a small section of the forward chamber. The red dotted line indicates the entrance of the microchannels. Arrows indicate puncta that move retrogradely towards the forward chamber. **A**, bottom) When PFF-ATTO was added to the forward chamber, no examples of moving puncta were found within the 24 h timeframe. An example is shown of a few puncta observed 5 days after adding PFF-ATTO, but these puncta did not reach the reverse chamber. **B** Two examples of puncta moving in the directly seeded chamber. **C** The speed calculated for puncta within the microchannel moving in the retrograde direction (*n* = 9, 2 devices) compared with the speed of puncta moving within the seeded chamber (*n* = 17, 4 devices). Scale bars are 50 µm.

In subsequent experiments, the frame-rate of image collection was increased to determine the direction and speed of travel of the puncta in the microchannels. Images were collected for 10 min with an interval of 5 s, and this was repeated every 3 h for 24 h. When PFFs were seeded in the reverse chamber to assess retrograde transport, moving puncta were observed in 2 of 12 channels (2 devices, one in each). 9 puncta were detected, all moving in the retrograde direction, of which 4 puncta were observed exiting the microchannel into the forward chamber (Fig. 3A, kymograph 1 & 2). For the other 5 puncta, the overall 10-minute timeframe of the time-lapse was not long enough to confirm their exit from the microchannels, but their absence in subsequent movies suggests that they were transported across the microchannel barrier as well. The average movement speed of puncta within the microchannels (when moving undisturbed) was 1.11 ± 0.13 µm/s (Fig. 3C), which is in line with the speed reported for retrograde transport of fibrils by Gribaudo et al.^26^ (1.3 ± 0.8 µm/s). This is also similar to the average speed as measured in the seeded chamber of small puncta moving undisturbed (1.25 ± 0.17 µm/s; Fig. 3B, C). When PFFs were seeded in the forward chamber, no puncta were observed moving in the anterograde direction in the first 24 h (2 devices). The only example of movement was observed after 5 days, but the puncta did not exit the microchannels into the reverse chamber (Fig. 3A, kymograph 3). Taken together, this suggests that transport of PFFs is primarily driven by retrograde intracellular transport.

**Fig. 4:**
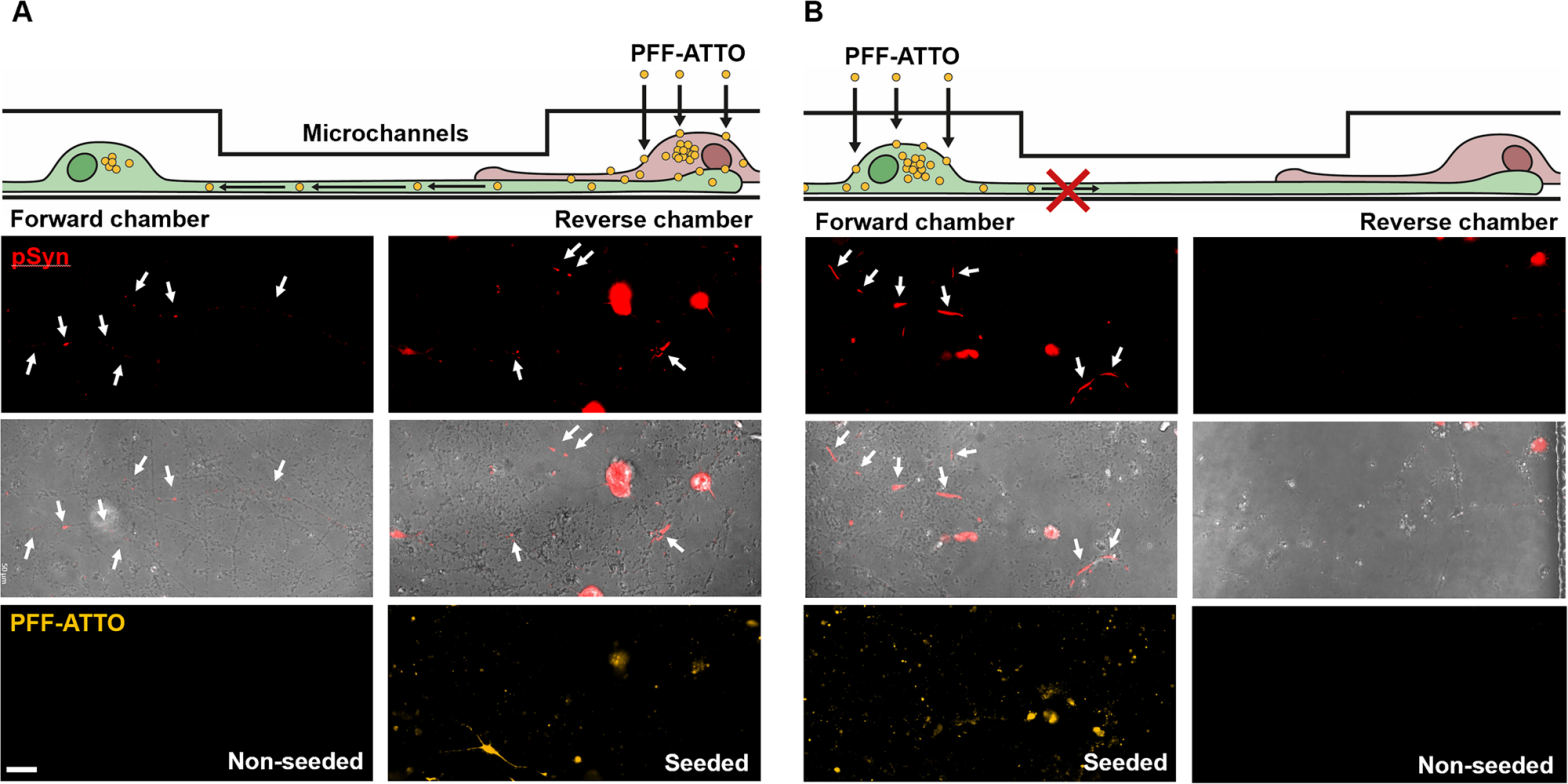
PFF-ATTO seeded in the reverse chamber resulted in αSyn pathology formation in both chambers, whereas seeding into the forward chamber only led to pathology formation on the seeded chamber. **A** Schematic of the PFF-seeding and retrograde transport. Below: immunostaining against pSer129-αSyn (pSyn, red) to identify αSyn pathology, pSyn overlayed with a phase microscopy image and ATTO-positive staining (yellow). Arrows indicate examples of pathology. The arrows indicate pSyn-positive pathology. **B** Schematic showing PFF-ATTO added to the forward chamber, but no transport is taking place. No αSyn pathology was found in the reverse chamber. 2 devices per condition. Scale bar is 50 µm.

To test if the PFF-ATTO transported retrogradely from the reverse to the forward chamber led to pSer129-positive structures, the devices were then incubated for 3 weeks, prior to staining. Although pathology levels were generally low, clear examples were observed in both seeded and unseeded chambers (Fig. 4A). When PFF-ATTO was seeded in the forward chamber, however, examples were only found in the seeded chamber, but not the reverse (unseeded) chamber (Fig. 4B). This further suggests that retrograde spread is the main mechanism of propagation in these experimental conditions.

### pSyn pathology increases over time in cultures directly seeded with PFFs

**Fig. 5:**
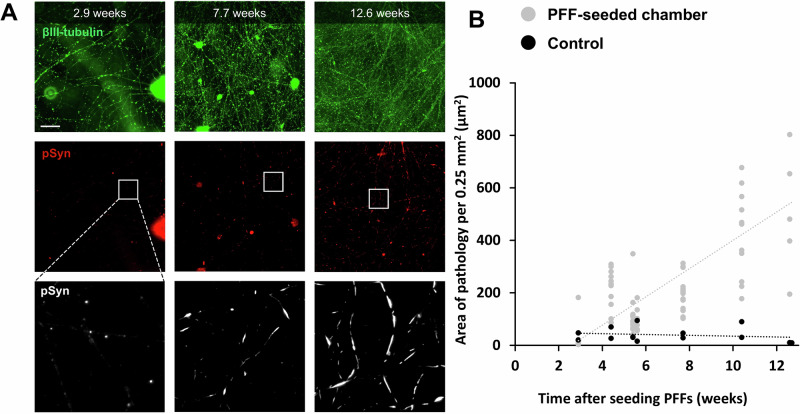
Pathology levels increase with time after seeding PFFs. Human AST18 mDA neurons were plated into microfluidics devices and allowed to mature until Day 65–70. At this point PPFs were seeded into the chamber and culturing was continued. Devices were fixed and prepared for immunostaining at different Days. The extent of Lewy-like pathology in both chambers was quantified by staining for phosphorylated αSyn at serine-129. Additionally, immunostaining of βIII-tubulin was used to only quantify pSer129- αSyn staining located on neurites. **A** Immunohistochemistry images showing the neurons stained with βIII-tubulin (green) and the development of pSyn pathology (red) over time. The bottom images show a zoomed-in version of the white squares. The number of weeks after seeding PFFs in the forward chamber is shown at the top of each set off images. The total area of pathology increases as well as the number of fragments. Furthermore, the shape of the fragments become more and more elongated and previously separated fragments start to blend together forming elongated structures. The scale bar is 100 µm. **B** Graphs showing the area of pathology per 0.25 mm^2^ in µm^2^ over time (in weeks; *n* = 69 devices). The amount of pathology increased over time, while the control devices that did not receive PFFs showed low levels of pathology and no increase over time.

PFFs were seeded into the forward chamber at Day 65–70 of differentiation, as seeding PFFs at earlier time points did not produce significant levels of pathology. Cultures were fixed at different timepoints for immunofluorescence, between 20 and 88 days post PFF seeding (Fig. 5A; Supplementary Fig. 12), and then imaged using epifluorescence microscopy. At 20 days (2.9 weeks) post PFF seeding, no significant pathology was observed when compared to the unseeded control condition (mean difference = 2.098, *p* = 0.130; Fig. 5A, first column). At 31 days (4.4 weeks) after seeding PFFs, slightly elongated pSyn-positive features started to be observed along the neurites in the forward chamber, with significantly higher levels of pathology than control experiments (mean difference = 167.859, *p* = 8.284e-7).

Increasing PFF incubation times dramatically increased pathology in the forward chamber, with a strong increase in both the number of pathological fragments per unit of area (0.25 mm^2^) (PFFs: y = 12.941x - 16.656, F(1,67) = 56.127, *p* = 1.983e-10, R² = 0.456; Control: y = −0.135x + 12.371, F(1,12) = 0.044, *p* = 0.838, R² = 0.004) and the total area (µm^2^) of pathology per unit of area (0.25 mm^2^) (PFFs: y = 53.828x - 137.706, F(1,67) = 68.460, *p* = 7.715e-12, R² = 0.505; Control: y = −1.492x + 50.331, F(1,12) = 0.401, *p* = 0.539, R² = 0.032) (Fig. 5B; Supplementary Fig. 15A left). This time-dependent increase in pathology was not dependent on the total area of neurites, as the latter did not change significantly after 4 weeks (F(1,61) = 2.180, *p* = 0.145, R^2^ = 0.035). We speculate this was due to neurites latching onto axon bundles over time, rather than spreading over unoccupied space. Therefore, data is presented both as the area of pathology normalised to the field of view of the objective used to image the cultures and as a percentage of the neurite area (Supplementary Fig. 15B, left), both show similar trends. To test the overall increase in pSyn pathology, data obtained from experiments with PFF incubation times above 4.4 weeks were combined. A Mann Whitney U test showed a significant increase in area of pathology (µm^2^/0.25 mm^2^) when compared to the unseeded control condition (mean difference = 191.317, *p* = 1.870e-6). The number of pathology fragments per 0.25 mm^2^ also showed a significant increase (mean difference = 67.154, *p* = 7.436e-7).

### Retrograde spread of pSyn pathology from one chamber to the other is more efficient than anterograde spread

Focussing on the formation of pSyn pathology over timeframes of around 7 weeks post PFF seeding, we investigated the anterograde and retrograde propagation of pSyn pathology along axons projecting respectively from the forward to the reverse chamber and vice versa.

**Fig. 6:**
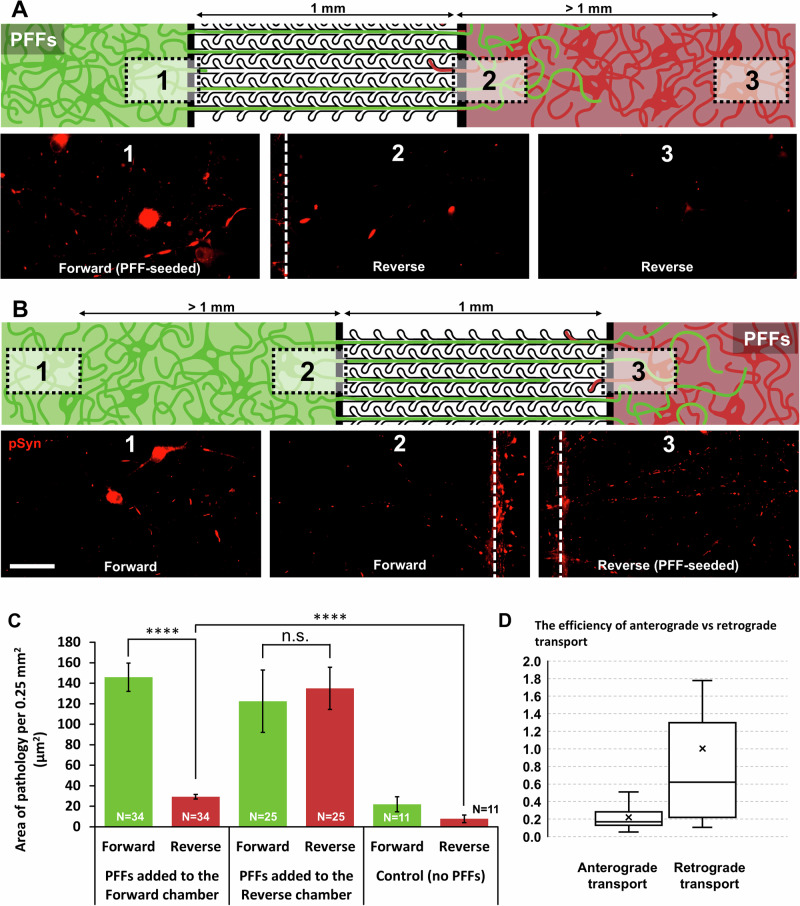
Anterograde spread is slow and levels are low, while retrograde spread is much more efficient. **A** When PFFs are seeded in the forward chamber, not much pSyn pathology (red) is found close to the microchannels, and the pathology level decreases further away from the channels. **B** When PFFs are added to the reverse chamber, pSyn pathology is present throughout the forward chamber, both close to the microchannels as well as more than 1 mm away. Scale bar is 100 µm. **C** Quantification of the results. When seeding PFFs in the forward chamber, pathology levels in the reverse chamber are low, but significantly higher than control levels (*n* = 34 devices, control: *n* = 11). When seeding into the reverse chamber, levels in the forward chamber are high (*n* = 25). **D** The ratios of transport were plotted by dividing the level of pathology in the unseeded side by the level in the seeded side for both types of experiments. This provides an estimation of the difference in efficiency between anterograde and retrograde transport/transfer. *p ≤ 0.05; **p ≤ 0.01; ***p ≤ 0.001; ****p ≤ 0.0001.

When PFFs were seeded in the forward chamber, overall levels of pathology in the reverse chamber were much lower than in the forward chamber (Forward: 145.9 ± 13.8 µm^2^/0.25 mm^2^, Reverse: 29.3 ± 2.4 µm^2^/0.25 mm^2^, mean difference = 116.6, *p* = 3.384e-12) and fragments were increasingly rare further away from the microchannels (Fig. 6A, ROIs 2&3). However, overall levels in the reverse chamber were significantly higher compared to the unseeded control condition (Area: mean difference = 21.518, *p* = 3.564e-6; Nr. of fragments: mean difference = 6.075 fragments/0.25 mm^2^, *p* = 8.418e-6; Fig. 6C; Supplementary Fig. 13A for nr. of fragments/0.25 mm^2^; Supplementary Fig. 13B for area of pathology as % of neurite area) suggesting that anterograde pathology spread did take place.

When PFFs were seeded in the reverse chamber, however, levels in the non-seeded forward chamber were relatively high and numerous examples of pSyn pathology were found even in areas that were over 1 mm distant from the microchannels (Fig. 6B, ROI 1). This is in line with in vivo studies^27–29^ identifying retrograde transport as the primary mechanism of pathology propagation. Quantifying overall pathology levels confirmed this observation with average levels in both chambers much exceeding control levels. (Seeded reverse chamber - Area: mean difference: 100.5, *p* = 0.00158 µm^2^/0.25 mm^2^; Nr. of fragments: mean difference = 33.1 fragments/0.25 mm^2^, *p* = 0.000298; Non-seeded forward chamber - Area: mean difference: 127.2 µm^2^/0.25 mm^2^, *p* = 4.182e-6; nr. of fragments: mean difference: 43.5 fragments/0.25 mm^2^, *p* = 3.509e-6; Fig. 6C; Supplementary Fig. 13A, B).

To compare the efficiency of anterograde and retrograde pathology propagation, we plotted the ratios of pathology area in the unseeded side over the level in the seeded side for both types of experiment (PFFs in forward chamber or PFFs in reverse chamber), showing that retrograde spread was approximately 4.5 times more efficient than anterograde spread for PFF incubation times under 8 weeks (Fig. 6D; mean difference: 0.78, *p* = 0.000161), a difference that increased with longer incubation times.

### pSyn pathology in the reverse chamber is weakly propagated to neurons from forward originating axons

In contrast to the directly seeded chamber, the level of pathology in the reverse chamber did not increase significantly over time (Area: PFFs: y = 0.0224x + 30.051, F(1,66) = 0.000978, *p* = 0.975, R² = 0.00148, Control: y = 24.531x - 1.733, F(1,16) = 1.180, p = 0.294, R² = 0.0687; Supplementary Figs. 14A, 15A, B), suggesting that the pathology propagated along the forward-originating axons did not significantly spread to neurons in the reverse chamber within the 3 months’ timeframe of our experiments. Only when selecting a region of interest within 400 µm from the microchannels, could an increase in the pathology levels be observed (PFFs: y = 7.821x + 3.325, F(1,67) = 39.207, p = 3.097e-8, R² = 0.369, Control: y = −0.260x + 11.500, F(1,10) = 0.100, p = 0. 758, R² = 0. 00994; Supplementary Figs. 14B, 15C, D). Forward-originating axons were very sparse beyond this region, so this difference suggests that pathology reaches the reverse chamber primarily through intracellular spread. However, low levels of transfer to neurons in the reverse chamber could not be excluded.

**Fig. 7:**
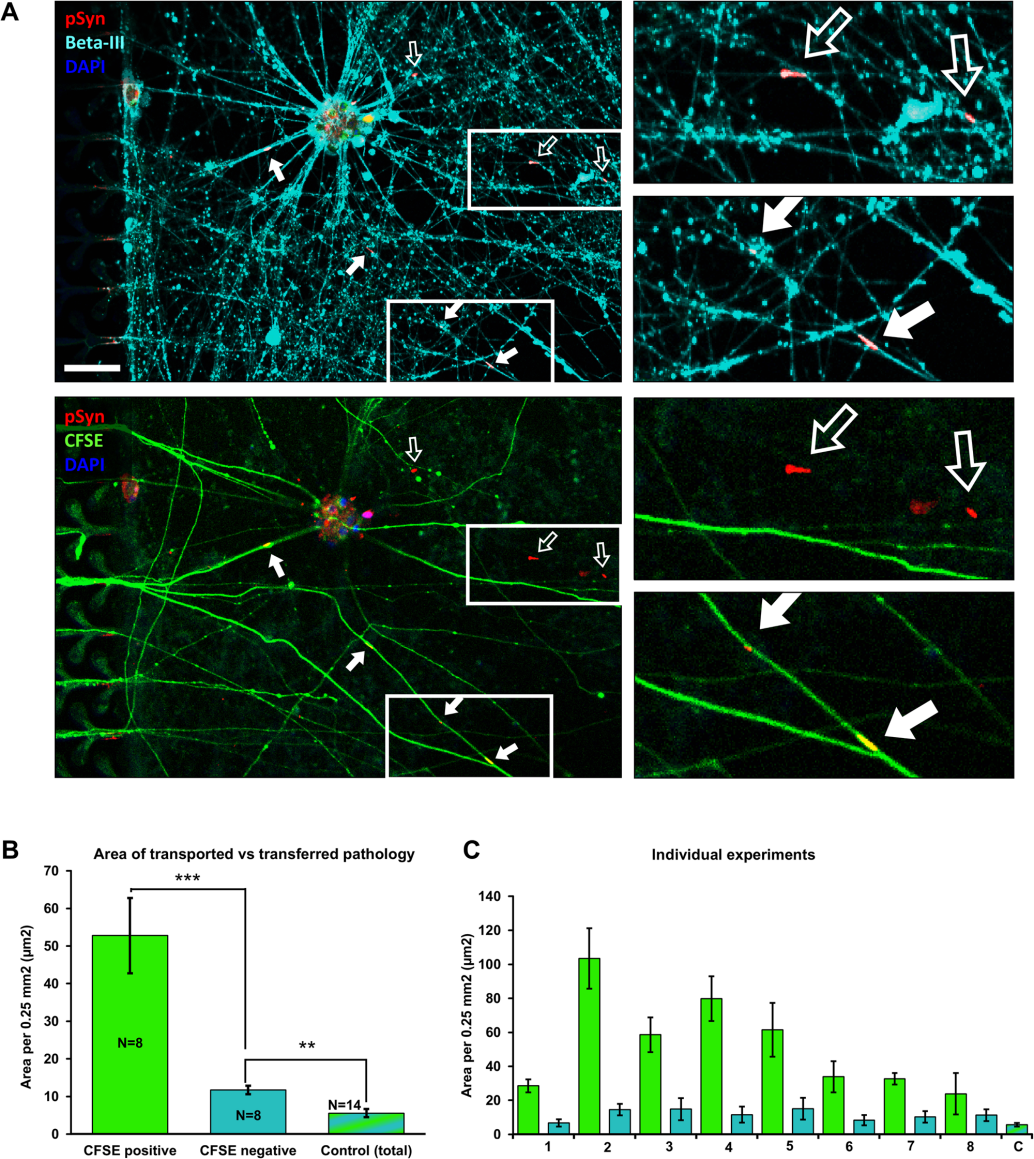
Anterograde transfer occurs in very small amounts. **A** top image: pSyn overlayed with βIII-tubulin. Bottom image: pSyn overlayed with CFSE. Closed arrows indicate examples of pSyn pathology that are CFSE-positive. Open arrows indicate example of CFSE-negative pathology. The scale bar is 50 µm. **B** Graph of the average area for the CFSE-positive and CFSE-negative pathology (*n* = 8 devices) and the combined pathology in control conditions (*n* = 14). **C** The values of CFSE-positive and CFSE-negative pathology levels for all individual experiments (1–8). The average total control level (**C**) is included for reference.

Having established propagation of pathology through forward originating axons, we investigated the extent to which pathology could spread to neurons in the reverse growth chamber. For this, cells were plated in both chambers and PFFs seeded only in the forward chamber. We applied and optimised methodology to distinguish between axons present in the reverse chamber that originated from the forward chamber. Immediately prior to fixing and staining the cultures, carboxyfluorescein succinimidyl ester (CFSE) dye was applied only to the forward chamber, using the same fluidic isolation protocol as per PFF confinement. CFSE is efficiently transported intracellularly within neurons and was therefore used to visualize forward-originating axons in the reverse chamber without staining neurons originally plated in the reverse chamber. The cultures were imaged and subsequently prepared for immunofluorescence using antibodies against pSer-129-αSyn and βIII-tubulin. By overlaying the CFSE image with the images taken after immunostaining, we created a mask to distinguish whether pSyn pathology was co-localised with forward or reverse originating neurites, thus allowing us to quantify CFSE-positive and CFSE-negative pSer-129-αSyn staining (also co-localised with βIII-tubulin) as a measure of transported versus transferred pathology. Results from image analysis revealed that the majority of overall pathology in the reverse chamber was associated with CFSE-positive neurites (52.770 ± 9.996 µm^2^/0.25 mm^2^; Fig. 7A, closed arrows; Fig. 7B, C). However, low levels of CFSE-negative pSyn pathology that colocalised with βIII-tubulin were also found (11.745 ± 1.116 µm^2^/0.25 mm^2^; Fig. 7A, open arrows point to examples that are positive for βIII-tubulin, but CFSE-negative; Fig. 7B, C). In contrast, pathology levels in two unseeded control devices for CFSE-positive neurites were 0.56 and 0.50 µm^2^/0.25 mm^2^. For CFSE-negative neurites the levels were 0.65 and 0.60 µm^2^/0.25 mm^2^. It must be noted that the intensity of the CFSE fluorescence signal in the reverse chamber decreased proportionally with the distance from the microchannels, therefore, only ROIs within 400 µm from the microchannels were used for this analysis. Taken together, the data suggest that pathology found in the reverse chamber was primarily due to anterograde transport along forward-originating axons, but that cell-to-cell transfer also occurred at a low level above controls (Fig. 7B, C).

To strengthen the statistical evidence of this observation, we quantified the levels of background pathology in the same area close to the microchannels in 12 additional control devices (no PFFs) not treated with CFSE. We therefore quantified the total levels of pathology to test against the CFSE-negative levels. Note that this approach decreased the chance of finding a false positive result. The area of CFSE-negative pathology in the PFF-seeded devices was 11.745 ± 1.12 µm^2^/0.25 mm^2^ and the control level was 5.557 ± 1.062 µm^2^/0.25 mm^2^ (mean difference: 6.188, p = 0.000956; Fig. 7B). A similar result was found for the number of pathology fragments which was 4.399 ± 0.436 fragments/0.25 mm^2^ for PFF-seeded devices and 1.792 ± 0.295 fragments/0.25 mm^2^ for control devices (mean difference: 2.606, *p* = 0.000637; Supplementary Fig. 13C; see 13D for area as % of neurite area). This suggest that anterograde spread of pathology to neurons in the reverse chamber does indeed occur at low levels.

## Discussion

A current prevailing model of early PD states that pathology likely starts in the periphery such as the gut or olfactory bulb, and spreads to multiple brain regions, notably the *substantia nigra* and frontal cortex. Exactly how αSyn pathology spreads between brain regions has been an active area of investigation. However, previous in vivo and in vitro studies did not provide a unified view on whether the spread is primarily driven by anterograde propagation, retrograde propagation, or both. Potential reasons for this discrepancy may be found in the lack of physiological relevance of the in vitro disease model and the challenges found by in vitro technologies when replicating in vivo complexity. Indeed, the ability to achieve complete unidirectional axon growth in vitro provides a tool that enables a mechanistic and robust analysis of early PD conditions.

To investigate this, we validated novel device layouts and methodologies for long-term cultures of hiPSC-derived neurons while maintaining unidirectional axonal topology and facilitating sufficient maturation of the neuronal cultures. Through miniaturized and live-cell image-based techniques, we differentiated between anterograde pathology spread and cell-to-cell pathology transfer, showing that retrograde transport of αSyn PFFs was at least 4 times more efficient than anterograde PFF transport. Furthermore, we found that a small but significant amount of pSyn pathology did spread anterogradely to neurons in the non-seeded chamber. Overall, our experimental platform can offer a powerful system to mimic early stages of PD development and progression. Our results are in line with in vivo studies that show retrograde spread of pathology is more prevalent and efficient than anterograde propagation^27–31^. This suggests that our model provides a better approximation to physiological conditions than previous in vitro models and would thereby be suitable to study potential PD treatments that act on αSyn aggregation and spread.

A fundamental aspect of this study was to demonstrate the robustness of our platform in achieving unidirectional axonal growth, a considerable challenge when culturing hiPSC neurons over a period of several months. Quantification of unidirectionality of ≤1% was achieved from experiments with cells cultured in one chamber only at a time, identifying microchannel length, time and shape of the pattern as the key parameters to achieve robust axonal unidirectionality. However, it is important to point out that in all edge-guided axonal strategies, one cannot exclude that when culturing neurons in both chambers, a forward-originating axon that had reached (or is found in proximity of) the reverse chamber could be used as an “anchor” by a reverse-originating axon, “latching-on” and growing along its path, thereby reaching the forward chamber. The experiment performed using dual viral infection of mouse cortical neurons (expressing EGFP or m-cherry) cultured in both chambers, confirmed this phenomenon rarely occured.

A further confirmation of the high-fidelity axonal unidirectionality obtained from our microfluidic platform is evidenced by the stark asymmetry in the transport of labelled PFFs across the microchannels (Fig. 3), as no example of anterograde PFF transport was found. In contrast, anterograde spread of αSyn pathology could be observed for extended culture periods only, with staining primarily localized close to the microchannels in the reverse chamber, which increased over time (Supplementary Fig. 14B, right; Supplementary Fig. 15C, D, right). Taken together these are demonstrations of the capabilities of the platform and methodologies produced in this work, allowing the study of anterograde/retrograde spread of endogenous αSyn aggregates, making it distinguishable from pathology initiated by transported PFFs.

The levels of PFF-induced pathology were relatively low, which was partly due to the time required to achieve a mature neuronal phenotype and to the type of fibrils. In our experiments, a period of 70 days of neuronal differentiation was required before seeding PFFs in order to obtain consistent and repeatable pSyn pathology. Furthermore, considering the higher surface to volume ratio of microfluidic devices with respect to well plate systems, one can expect a higher quantity of fibrils to adhere to the walls of the channels, these being prone to surface adsorption. We chose to use fibrils made from WT human synuclein, produced in mammalian cells. WT human synuclein has a lower misfolding and seeding propensity than fibrils made of A53T synuclein^4,57^. However, we wanted to use WT human synuclein fibrils to stay as close as possible to the form found in most patients.

When imaging ATTO-PFFs in microfluidic culture chambers using confocal microscopy, we observed two behaviours. In some cases, puncta that appeared during the 24 h time lapse were often followed by more puncta along the same section of the axon (e.g., Supplementary Video 2), indicating an accumulation of labelled-fibrils, due to transport within the neurite. These puncta became stationary along the neurites, which showed signs of swelling (i.e., puncta growing progressively wider than the neurite), which may account for their immobility. In other cases, over the 24 h time lapse, we observed puncta moving along neurites towards and into the cell soma, slowly accumulating in the soma, which may be compatible with retrograde transport.

In the current study, we focussed on the intracellular spread of αSyn pathology in mDA neurons and potential pathology transfer between neurons. Further advancement of this model and experimental platform could be gained by creating a co-culture, for example, with astrocytes and microglial cells, as these cell types could play a major contribution to the spread of extracellular pathological synuclein aggregates within the brain. For example, glial cells have been shown to be involved in PD pathogenesis both by their ability to internalise transferred αSyn and their role in inflammation^58,59^. In a recent study, hiPSC-derived mDA neurons were co-cultured with glial cells, pericytes and microvascular brain endothelial cells on a microfluidics chip to mimic the blood-brain barrier function^60^.

Combining these co-culture models with lab-on-chip methodologies will result in increasingly more refined models with in vivo-like physiological conditions, providing a useful tool to assess the interaction between αSyn pathology propagation and other mechanisms at play in PD pathogenesis, as well as a general tool for studying other proteinopathies and screening of potential disease-modifying treatments.

## Methods

### Human iPSC-derived midbrain dopaminergic neuronal differentiation

The AST18 hiPSC line, derived from a patient with a triplication of *SNCA*, encoding αSyn^44^, was used for the differentiation of midbrain dopaminergic (mDA) neurons. The collection of fibroblasts to generate the iPSC lines was previously published^44^ and was approved by the Royal Free Hospital and Medical School Research Ethics Committee with Project Reference Number: 07/H0720/161. Differentiation was achieved according to the protocol described by Chen et al.^17^ and available on protocols.io: 10.17504/protocols.io.bddpi25n. Self-renewing hiPSCs were lifted with EDTA (0.5 mM), counted and plated at 40,000 cells/cm^2^ on Laminin-111-coated plates (L111, 5 μg/ml, Biolamina) in 50% Neurobasal™ medium (Thermo Fisher Scientific), 50% DMEM/F12 (Thermo Fisher Scientific), B27 (1:50, Thermo Fisher Scientific), N2 (1:100, Thermo Fisher Scientific), L-glutamine (2 mM, Thermo Fisher Scientific), Sonic hedgehog (SHH-C24II, 600 ng/ml, R&D), CHIR99021 (1 μM, Miltenyi Biotec), SB431542 (10 μM, Tocris), LDN193189 (100 nM, Stemgent) and Y27632 (10 μM, Tocris). The culture medium was exchanged on Day 2 with the above medium without Y27632. On Day 4 and Day 7 cells were fed with medium consisting of 50% Neurobasal, 50% DMEM/F12, B27 (1:100), N2 (1:200), L-glutamine (2 mM), SHH-C24II (600 ng/ml), CHIR99021 (1 μM), SB431542 (10 μM), and LDN193189 (100 nM). On Day 9 cells were fed with the above medium supplemented with FGF8b (100 ng/ml, R&D) and heparin (1 μg/ml, Sigma). On Day 11 cells were lifted using Accutase (Sigma) and re-plated at 800,000 cells/cm^2^ on L111-coated plates in Neurobasal medium supplemented with B27 (1:50), L-glutamine (2 mM), BDNF (20 ng/ml, Peprotech), GDNF (10 ng/ml, Peprotech), ascorbic acid (0.2 mM, Sigma), FGF8b (100 ng/ml), heparin (1 μg/ml), and Y27632 (10 μM). Cells were fed on Day 14 with Day 11 medium without Y27632. On Day 16 cells were lifted with Accutase and cryopreserved in PSC Cryopreservation Medium (Thermo Fisher Scientific) for later use^50^. The identity of the mDA progenitor cells was confirmed by immunostaining for the mDA markers LMX1A, FOXA2, tyrosine hydroxylase (TH), and the pan-neuronal marker βIII-tubulin. Briefly, cells were fixed with 4% formaldehyde for 20 min and washed three times in PBS prior to addition of blocking buffer (0.1% Triton X-100, 2% goat serum or donkey serum in PBS). Primary antibodies TH (1:1000, rabbit, Millipore), βIII-tubulin (1:1000, TuJ1, mouse IgG2a, R&D), LMX1A (1:2000, rabbit, Millipore), and FOXA2 (1:100, goat, Santa Cruz) were incubated with fixed cells overnight at 4 °C and then washed three times in PBS with 0.1% Triton X-100. Secondary antibodies (Thermo Fisher Scientific) in blocking buffer were incubated with cells for 2 h, washed three times in PBS with Triton X-100 before imaging on an Olympus IX51 inverted microscope.

### Plating and culture of human iPSC-derived midbrain dopaminergic neurons in microfluidic devices

Day 16 mDA progenitor cells were thawed and grown for seven days in a culture plate coated with poly-L-ornithine (0.01%, Merck) and laminin-111 (LN-111, 5 µg/ml, Biolamina), after which they were lifted using Accutase (Merck) and plated into microfluidic devices at Day 23. The neural culture was maintained in Neurobasal medium (ThermoFisher) with added L-glutamine (ThermoFisher) and B27 supplement without vitamin A (ThermoFisher). Additional supplements were: dibutyryl cyclic AMP (0.5 mM, Sigma), DAPT (1 µM, Tocris), ascorbic acid (0.2 mM; Merck), brain-derived neurotrophic factor (BDNF, 20 ng/ml, Peprotech) and glial cell line-derived neurotrophic factor (GDNF, 10 ng/ml, Peprotech). From Day 40 onwards, DAPT was omitted from the medium. When plating cells, Y27632 (Y2, 10 µM, Tocris) was added to the medium. Twice a week, half of the medium was removed and replaced by fresh medium. Medium was always added first to one well to allow the fresh medium to superfuse the entire culture chamber before the second well was also filled. Devices were kept at 37 °C, 5% CO_2_ and 95% humidity.

Cultures were maintained for 10–24 weeks after plating in microfluidic devices to assess potential time-dependent effects. A suspension of single cells was injected into the microfluidic devices, resulting in a homogenously distributed culture immediately after plating and increasingly clustered over the course of many weeks (Supplementary Fig. 16A). Typically, neurites started to grow after a few days and cell clusters were observed after about 3 weeks. After 2 months of culture in devices most neurons formed dense rosettes of various sizes, some of which reached the ceiling of the microfluidic chamber, forming column-like structures, connected by an intricate network of neurites. Neurites grew both on the coated coverslip and the polydimethylsiloxane (PDMS) walls (Supplementary Fig 16B). However, quantitative image analysis was performed only for cells and neurites growing on the flat coverslip surfaces.

### Microfluidic device fabrication

Microfluidic devices, consisting of two culture chambers separated by an array of microchannels were produced in polydimethylsiloxane (PDMS) (Sylgard 184, Dow Corning, US) using photo/soft-lithography techniques, as previously reported^61^. In short, two layers of respectively 4 µm (forming the microchannels) and 90 µm (forming the chambers) thickness were fabricated onto a silicon wafer using SU-8 photoresist (SU8 3005 and SU8 3035 respectively, MicroChem, US). Photoresist was exposed to UV-light through photomasks (JD PhotoData) designed to create the patterned microchannels and chamber layouts. The silicon master was then salinized by vapour deposition of 1H,1H,2H,2H-perfluorooctyl-trichlorosilane (Merck, UK) for 1 h and placed in a glass petri dish. PDMS (base to curing agent ratio 1:10) was poured onto the silicon master and degassed in a vacuum desiccator prior to curing at 80 °C overnight. The PDMS devices were removed from the petri dish and cut to the desired size. A 6 mm punch (Integra Miltex) was used to create open wells at each end of a chamber to provide fluidic access. The devices and glass coverslips (VWR, UK) were cleaned prior to bonding using oxygen plasma. Devices were then placed in an oven at 80 °C overnight to strengthen the bonding process. Next, devices were flooded with oxygen plasma to make their surface hydrophilic and sterile. The devices were then coated with poly-L-ornithine and recombinant laminin 111 (Biolamina, LN111) and stored in the fridge for later use.

### αSyn Preformed Fibril (PFF) formation and characterization

Human *SNCA* cDNA cloned into the pMKC451 plasmid was transfected into Expi293 cells using Expi293fectamine (1956760, Gibco), according to manufacturer’s instructions. The culture was harvested 4 days after transfection by centrifugation (90 min, 4000 rpm, JS4.2 rotor in J6-MI Beckman) and filter sterilised through a 0.22 µm filter (Millipak Gammagold, Millipore) using a Sartobran P 0.45 + 0.22 µm pre-filter (Sartorius Stedim). Lysate was diluted with an equal volume 20 mM Tris (T1503, Sigma) / HCl (20252.335, VWR Chemicals) pH 8.0 and was passed over a 2 x 5 ml HiTrap Q FF column (17-5156-01, GE Healthcare). Bound protein was eluted with a gradient to 0.4 M NaCl (S/3160/60, Fisher Chemical) in 20 mM Tris/HCl over 20 column volumes. Fractions with α-synuclein were pooled, concentrated using a Centriprep 10 K, 10,000 NMWL spin filter (4305, Millipore) and buffer exchanged into 20 mM Tris/HCl pH8.0 using a HiPrep 26/10 desalting column (GE Healthcare). Anion exchange chromatography was repeated using a MonoQ 10/100GL column (GE Healthcare) and fractions with α-synuclein were pooled, concentrated and purified over a HiLoad 26/600 Superdex 75 column (GE Healthcare). Analysis throughout the purifications was performed by 4–12% NuPage Bis/Tris gel electrophoresis. The final pool was concentrated, filter sterilised (0.22 µm Millex GV, Millipore) to 7–10 mg/ml monomeric protein. The preformed fibrils (PFFs) were prepared by shaking the purified human α-synuclein monomer (7–10 mg/ml, 0.50 ml) in 1.5 ml BioPur Eppendorf vials at 1200 rpm, 37 °C for 10 days using a VorTemp56 incubator (Labnet). The resulting material was sonicated for 4 × 10 s at 2.5 mg/ml in 1.5 ml BioPur Eppendorf vial on ice using a Soniprep sonicator (MSE). The protein solution was aliquoted and frozen on dry ice and stored at –80 °C in BioPur Eppendorf vials. The PFFs were directly compared with monomers in a previous paper by Chen et al. using human mDA neurons^17^.

### αSyn fibril preparation

N-terminal-cysteine (M1C) tagged human αSyn was expressed transiently in Expi293F cells according to manufacturer’s instructions (Thermo Fisher Scientific) and cultures were harvested after 5 days expression. Protein was captured from the supernatants using AEX chromatography (HiTrap Q FF, GE Healthcare) and gradient eluted at around 150 mM NaCl in 20 mM Tris/HCl pH8.0, AEX was repeated on MonoQ 10/100GL and protein pool was purified on a HiLoad 26/600 Superdex 75 column (GE Healthcare) in PBS pH7.4. Monomers were stored at −80 °C.

M1C h αSyn was conjugated to ATTO550-maleimide (30730, Sigma) following manufacturer’s protocol. To generate fluorescent fibrils, tagged αSyn was mixed with non-tagged αSyn at a molar ration of 1:21. Sealed aliquots were incubated at 37 °C with continuous shaking at 1200 rpm for 7 days and stored at −80 °C. Fibrils were sonicated before each experiment in a Qsonica500 waterbath sonicator.

### αSyn PFF injection protocol

PFFs were seeded in only one chamber per device. Prior to and during PFF injection, the volume of the medium in the open wells of the opposite culture chamber was always kept at a higher hydrostatic pressure (greater liquid height level) than that in the wells of the injected fibrils, to ensure a constant flow across the microchannels towards the PFF seeded chamber. This condition avoided any potential injection of PFFs into the microchannels or the opposite chamber. The chamber to which PFFs were added was first emptied and then 45 µl of PFFs in medium was added to one of the open wells. After waiting for 10 min, 20 µl was removed from the same well and 20 µl of PFF solution was added to the other well and left for another 10 min. This procedure was repeated 3 times. Then the wells of the seeded chamber were filled equally, making sure that the fluid level did not exceed that of the opposite wells. From this point onwards this difference in fluid levels was maintained throughput the experiments to ensure fluidic isolation. Devices were tested for fluidic isolation between the two chambers. For this, the wells of the reverse chamber were filled with PBS and those of the forward chamber with PBS containing Calcein (50 µM). The PFF injection protocol was validated using a calcein solution instead of PFFs. Fluorescent imaging was used to demonstrate that no calcein reached the microchannels or the opposite chamber, confirming fluidic isolation between the chambers (Supplementary Fig. 5).

### Immunohistochemistry

For identification and quantification of the pSyn aggregates, an antibody against pSer-129-αSyn (anti-pSer129 produced in mouse, 1:300, 015-25191, Wako) was used. The pSer129 antibody ab51253 from Abcam was considered, but a comparison of both antibodies in the context of our platform showed that the Wako pSer129 antibody was better suited (Supplementary Fig. 17). Anti βIII-tubulin (produced in rabbit, 1:500, T2200, Merck) was used to visualise neurites. MAP2 polyclonal antibody (raised in chicken, 1:5000, PA1-10005, ThermoFisher) was used for staining dendrites. Markers for Lewy body-like pathology were: p62 (raised in rabbit, 1:200, ab91526, Abcam), HSP70 (chicken, 1:100, PA5-77828, ThermoFisher) and LC3B (rabbit, 1:200, PA1-46286, ThermoFisher). The secondary antibodies used were: Donkey anti-rabbit (488/350 nm; 1:200; ThermoFisher), Donkey anti-mouse (555 nm; 1:200; ThermoFisher) and Goat anti-chicken (633 nm; 1:200; ThermoFisher). Additionally, DAPI was used to stain the cell’s nuclei (350 nm; 1:1000). The intracellular fluorescent dye carboxyfluorescein diacetate succinimidyl ester (CFSE, ThermoFisher) was used to stain axonal growth from the forward chamber into the reverse chamber. By comparing pathology levels on CFSE-positive and CFSE-negative neurites in the reverse chamber a distinction could be made between transport of αSyn pathology and transfer. Transport is defined here as the intracellular movement of pathology along axons and transfer is used here for the transmission of αSyn pathology from one cell to another.

### Primary Hippocampal Neuron Culture

Primary hippocampal neurons were obtained from C57Bl6 mouse embryos. Briefly, E17 pregnant mice were anesthetized with isoflurane and sacrificed by decapitation, and hippocampi were dissected from the embryos in dissection buffer [HBSS without calcium or magnesium (ThermoFisher), 0.6% D-(+)-Glucose (Sigma), 20 mM Hepes (ThermoFisher)]. The hippocampi were then incubated for 20–30 min at 37 °C in papain dissociation solution [dissection buffer supplemented with 20 U/ml papain (Sigma P4762-100mg), 1 mg/ml DNase (Sigma DN25), 1 mM L-cysteine (Sigma C-7352), 0.5 mM EDTA (ThermoFisher 15575020)]. The dissociation buffer was then removed, and hippocampi were washed 3 times with 10 mL plating medium [Neurobasal Plus Medium, 2% B27 Plus Supplement, 2 mM GlutaMAX-I Supplement, 50 units/mL penicillin-streptomycin, 2.5% fetal bovine serum (ThermoFisher A3840002)]. After the last wash, the hippocampi were left in 1 mL of plating medium and then triturated gently with a P1000 pipette to break up the tissue. The cell suspension was then diluted in plating medium and 37,500 cells were plated per chamber in the microfluidic devices. Cells were then kept in a cell culture incubator, at 37 °C, 5% CO_2_, 95% humidity. 2 days after cell plating, lentivirus overexpressing mouse alpha-synuclein C-terminally tagged with eGFP or mCherry was added to the neurons with a multiplicity of infection of 2. Devices were imaged in an Opera Phenix plate imager (Perkin Elmer) every 15 min, using a 20x water-immersion objective, at 37 °C and 5% CO_2_. For each field, 2 confocal planes 2 micrometers apart were acquired.

### Imaging setup

An Axio observer Z1 (Zeiss) inverted microscope with LED illumination, connected to an Orca Flash 4.0 camera (Hamamatsu), was used for image acquisition. Devices were imaged using both phase and epifluorescence microscopy through a 20x objective and making use of the tiling function of the Zeiss Zen Blue software. For immunohistochemistry colocalization experiments for pSer129, p62, MAP2, LC3B and HSP70, confocal microscopy (Leica Microsystems SP8) or the Zeiss Axio observer Z1 with apotome.

For the live-imaging experiments, devices were imaged on an Opera Phenix Plus, spinning disk confocal microscope (Revvity) every 15 min, using a 20x water-immersion objective (Zeiss, NA 1.0) or every 5 s for 10 min sessions every 3 h using a 40x water-immersion objective (Zeiss, NA 1.1).

### Quantification of pSyn pathology by automated image analysis

A novel software routine in MatLab and a workflow for image processing (Supplementary Fig. 6) were developed to quantify pSyn pathology in microfluidic cultures, defined as an elongated fragment from pSer-129 staining. For both the forward and reverse chambers of each device, 10 images from the central area of the chamber (an area between 400 µm and 1600 µm away from the microchannels accounting for 70% of the available culture area; Supplementary Fig. 6A) were analysed. For some additional analyses, an area from the microchannels to 400 µm away was used, which is stated in the text for these instances. Images obtained from immunofluorescence readouts from pSer-129 and βIII-tubulin staining were loaded and converted into black-and-white images using a threshold algorithm (Supplementary Fig. 6B). Software parameters (threshold levels, pSer-129 fragment width, length and area) were optimised manually and then applied to all images. Only pSer-129 fragments that co-localised with βIII-tubulin were considered in the analysis. pSer-129 fragments that were below an arbitrary area (less than 8 µm^2^) were excluded in the quantification to reduce the influence of nonspecific staining. The software extracted the total areaa of pathology, the total number of fragments and the length of each fragment. Potential outliers were excluded from the analysis using the Grubb’s test and median values were calculated from the 10 images in each chamber. Results were averaged for a minimum of 6 devices. An adapted version of the software was developed specifically to quantify CFSE-positive and CFSE-negative pathology in the reverse chamber, as a measure of transported and transferred pathology. This was obtained by overlaying images from CFSE staining with images from immunohistochemistry in the reverse chamber. The software routines are available on request.

### Statistical analysis

To assess the change in pathology (area and number of fragments) over time, we used linear regressions. To test statistical differences between unpaired datasets, we used the non-parametric Mann Whitney U test. These tests were performed using R studio.

## Supplementary information

Supplementary Material

Supplementary Video1

Supplementary Video2

Supplementary Video3

Supplementary Video4

## Data Availability

The data that supports the finding of this study is available from the authors upon reasonable request.

## Abbreviations

PD: Parkinson’s disease
mDA: midbrain dopaminergic
aSyn: α -synuclein
pSyn: phosphorylated α -synuclein
PFF: preformed fibril
LB: Lewy body
hiPSC: human induced pluripotent stem cell
AST18: α-synuclein triplication 18
TH: tyrosine hydroxylase
MAP2: microtubule-associated protein-2
CFSE: carboxyfluorescein succinimidyl ester
ROI: region of interest

## Code availability

The data that supports the finding of this study is available from the authors upon reasonable request.
