# Supplementary material for: A high-fidelity microfluidic platform reveals retrograde propagation as the main mechanism of α-Synuclein spread in human neurons
Source: NPJ Parkinsons Dis. 2025 Apr 20;11:80. doi: 10.1038/s41531-025-00936-x (PMC12009960; doi:10.1038/s41531-025-00936-x)
Supplement: Supplementary file 1 — Supplementary Material [file 41531_2025_936_MOESM1_ESM.pdf]

Supplementary Information

A high-fidelity microfluidic platform reveals retrograde propagation as the main mechanism of α-Synuclein spread in human neurons

Rozan Vroman[a], Lorenzo de Lichtervelde[b], Karamjit Singh Dolt[c], Graham Robertson[a], Marco Kriek[d], Michela Barbato[c], Justyna Cholewa-Waclaw[c], Tilo Kunath[c,e] Patrick Downey[b] and Michele Zagnoni*[a]

[a] *Centre for Microsystems and Photonics, Department of Electronic and Electrical Engineering, University of Strathclyde, Glasgow, G1 1XW, UK*

[b] *UCB Biopharma, Chemin du Foriest, 1420 Braine-l'Alleud, Belgium*

[c] *Centre for Regenerative Medicine, Institute for Regeneration and Repair, The University of Edinburgh, Edinburgh, UK*

[d] *UCB Biopharma UK, Slough, UK*

[e] *Institute for Stem Cell Research, School of Biological Sciences, The University of Edinburgh, Edinburgh, UK*

** Corresponding author: michele.zagnoni@strath.ac.uk*

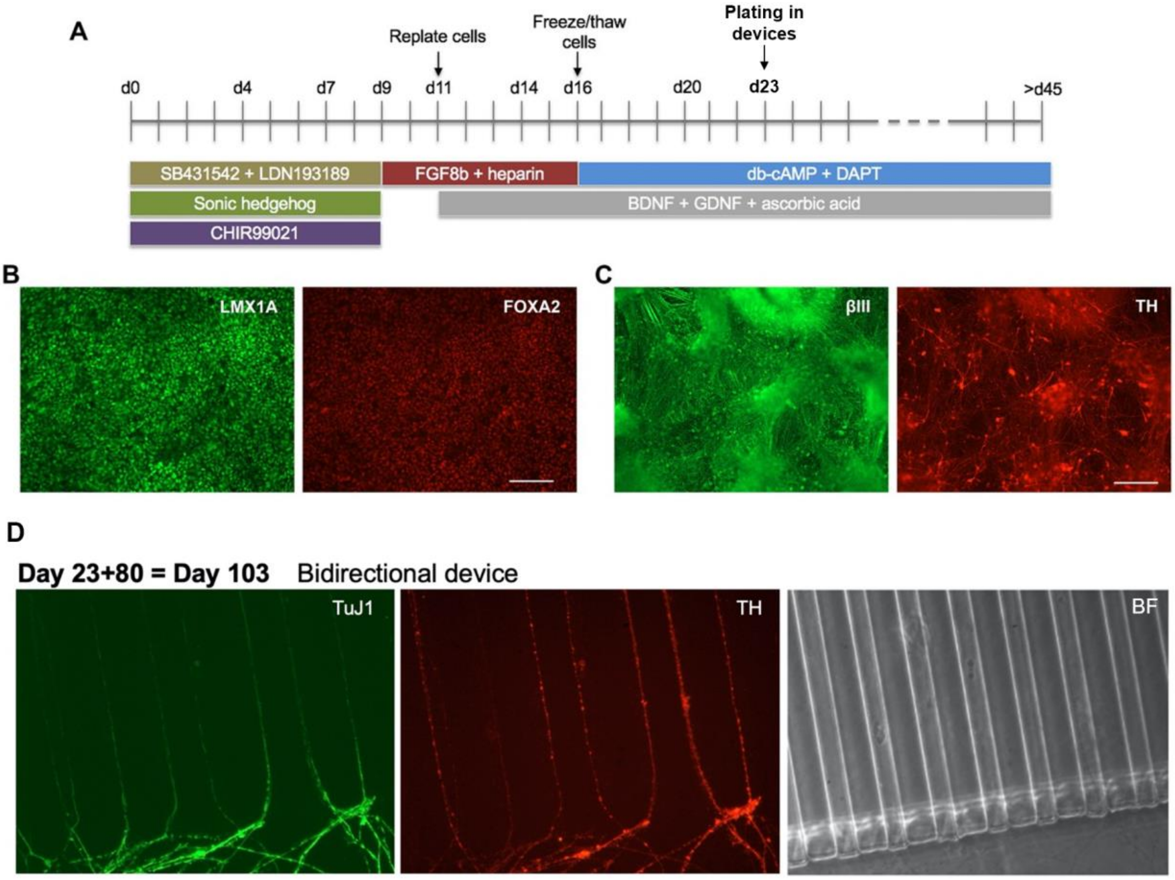

Supplementary figure 1 Characterisation of the hiPSC mDA cells and pSyn pathology. A) Overview of mDA differentiation protocol (see Methods for details). B) Day 16 mDA progenitor cells immunostained for LMX1A (green) and FOXA2 (red). The scale bar is 130 µm. C) Day 42 mDA neurons, 26 days after cryopreservation, immunostained for βIII-tubulin (green) and tyrosine hydroxylase (red). The scale bar is 220 µm. D) TuJ1 and TH expression in bidirectional devices. βIII-tubulin and tyrosine hydroxylase staining in bidirectional devices. A brightfield image of the device is shown at the bottom.

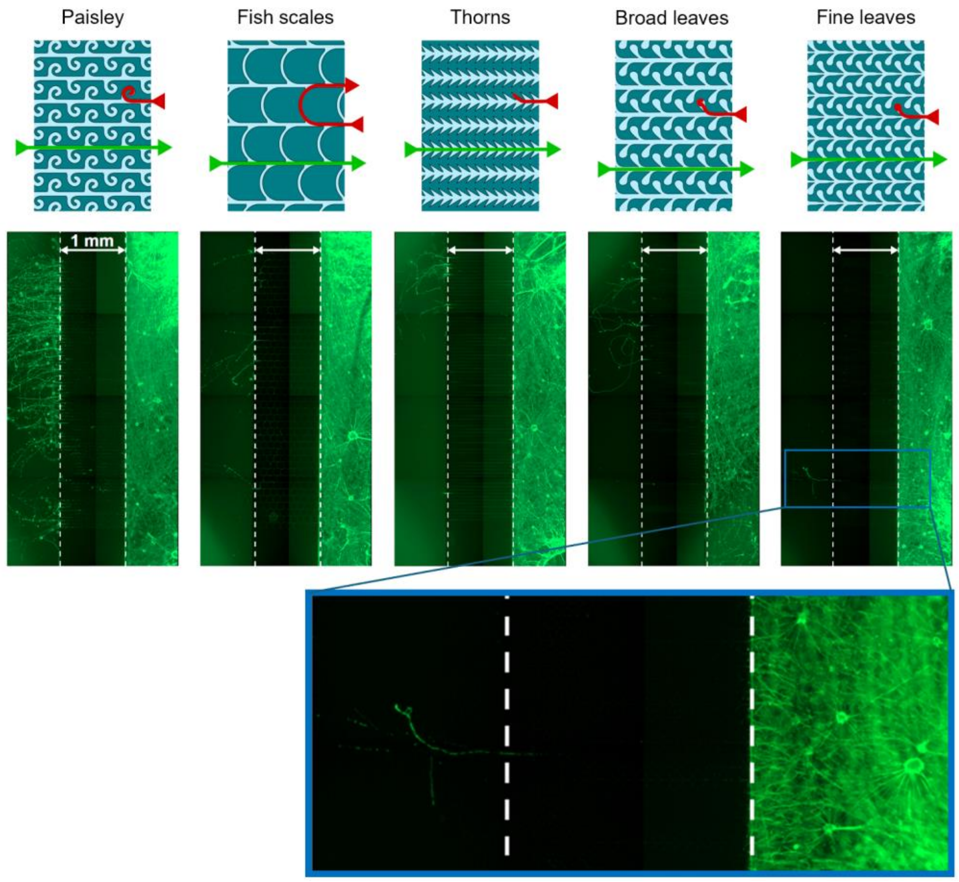

Supplementary figure 2 Extended images from Figure 1B with increased brightness and inset showing channel crossing in the “fine leaves” design.

A

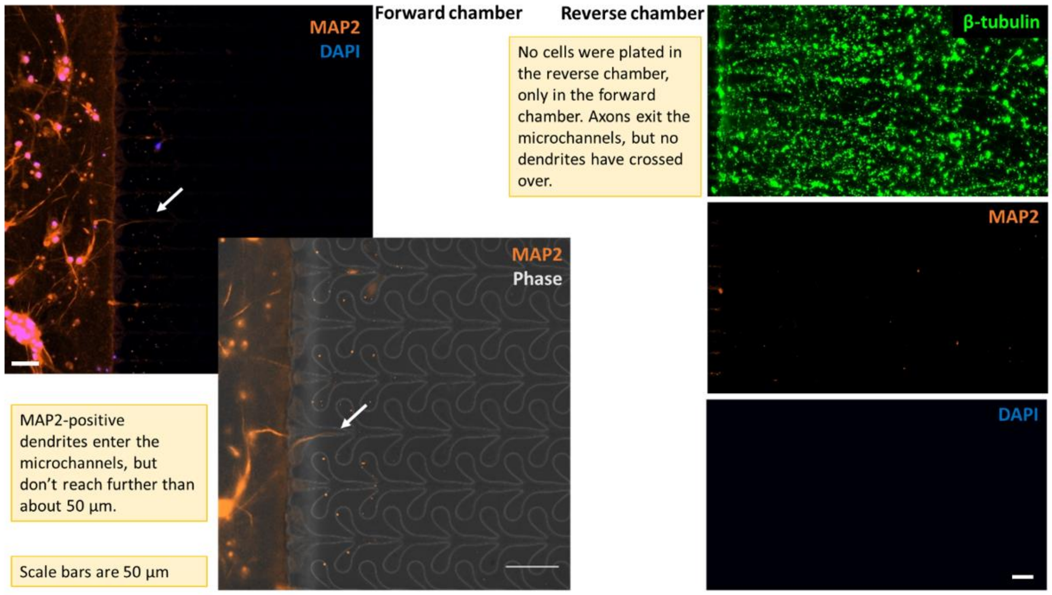

B

Whereas dendrites don't reach far into the microchannels, axons stained with β-tubulin cross from the forward to the reverse chamber. Here, a section of the microchannels is shown.

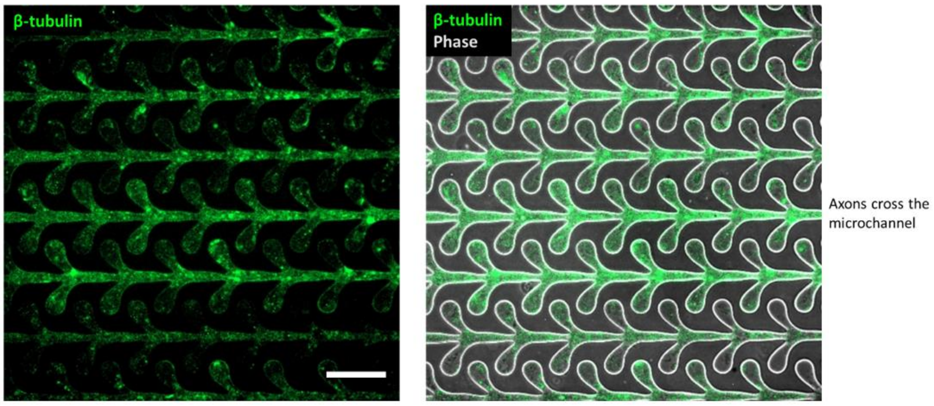

Supplementary figure 3 Dendrites in the forward chamber cannot reach far into the microchannels, while axons reach the reverse chamber with ease.

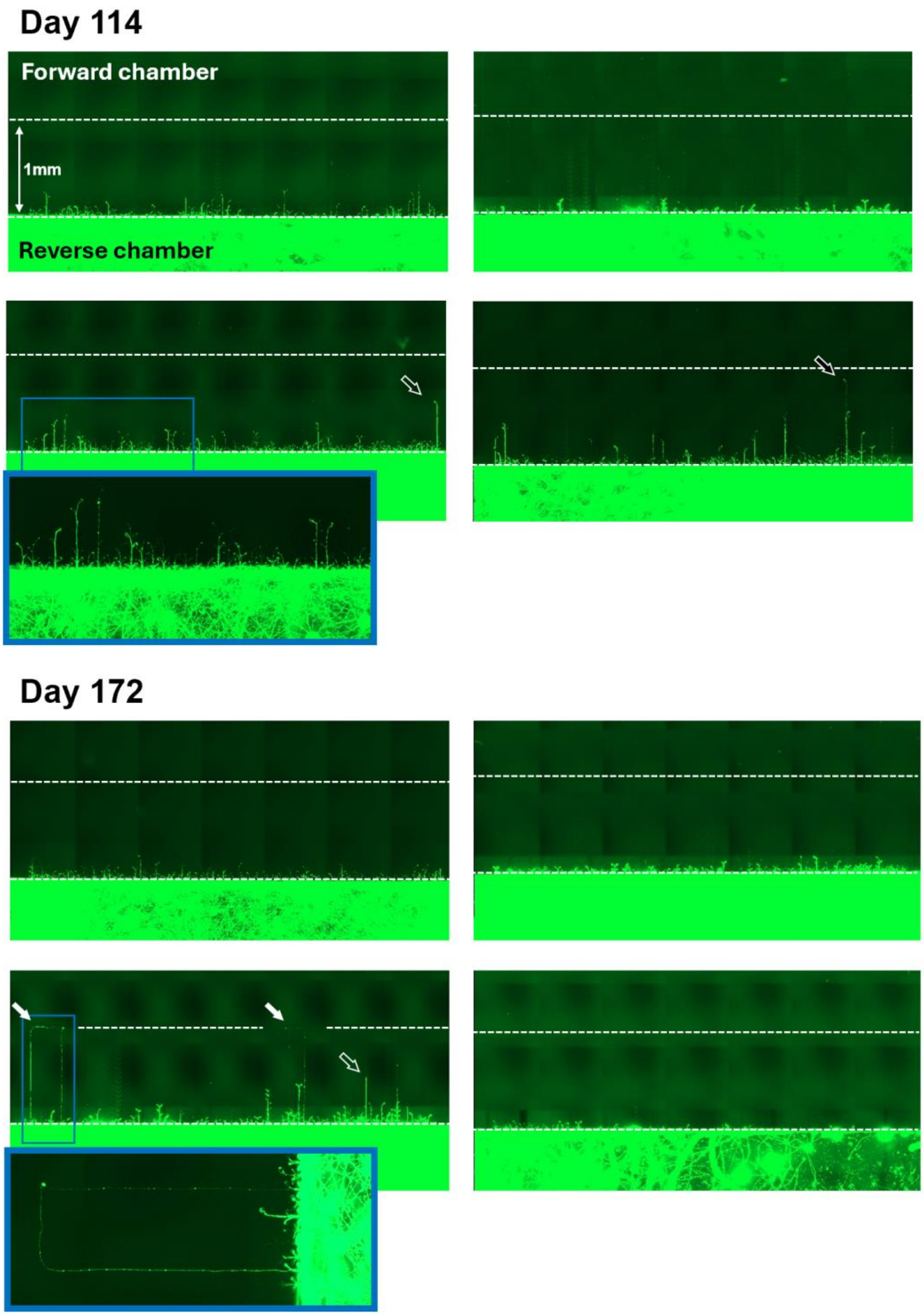

Supplementary figure 4 Testing the unidirectionality of the ‘fine leaves’ devices. At day 114 of differentiation, no axonal crossings were observed (4 devices). At day 172, 4 breaches of a microchannel are observed. The crossing axons are indicated by a filled arrow, whilst with open arrows axons are indicated that grew close to the forward chamber but did not reach it, remaining trapped in the leaf pattern.

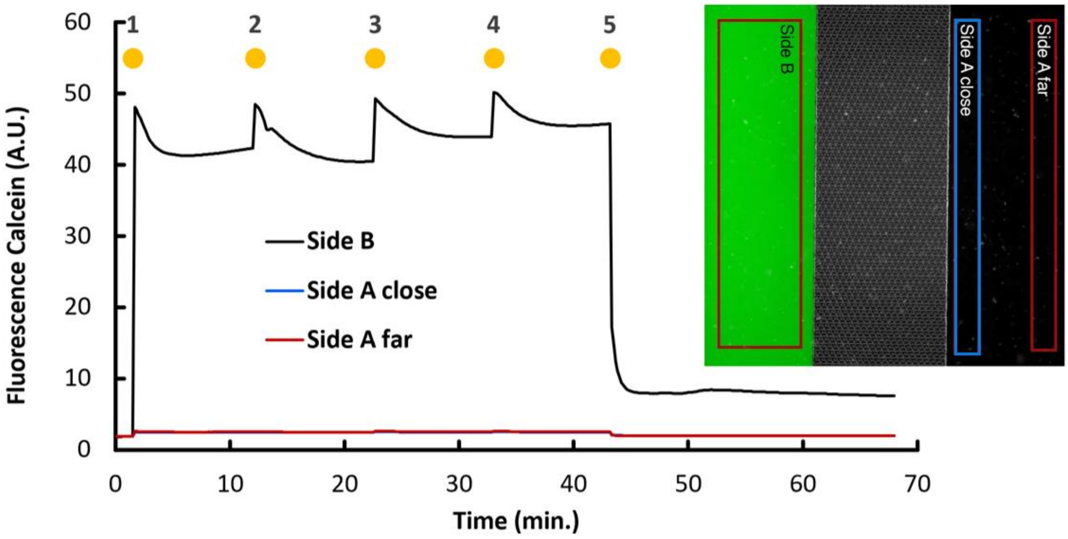

Supplementary figure 5 Graph showing the results of an experiment to test the fluidic isolation, which will prevent PFFs to enter the non-seeded chamber. Instead of PFFs in medium we used 50 µM calcein in PBS, but the same seeding protocol was used. The two wells in the Reverse chamber were filled with 50 µl PBS each to ensure a constant pressure towards the Forward side. 45 µl of the calcein solution was added to the top well of the Reverse side (point 1). After 10 minutes, 20 µl was removed from the top and 20 µl of fresh calcein solution was added to the bottom well (point 2). This was repeated 3 times with intervals of 10 minutes, switching wells each time (points 2, 3 & 4). Finally, both wells were topped up (point 5), making sure that the level of the Reverse wells was slightly higher. A slight increase in fluorescence due to reflection was observed in the Reverse chamber, but no diffusion of calcein. The inset shows the two chambers of the device (side A and B), connected by the 1mm long patterned microchannels.

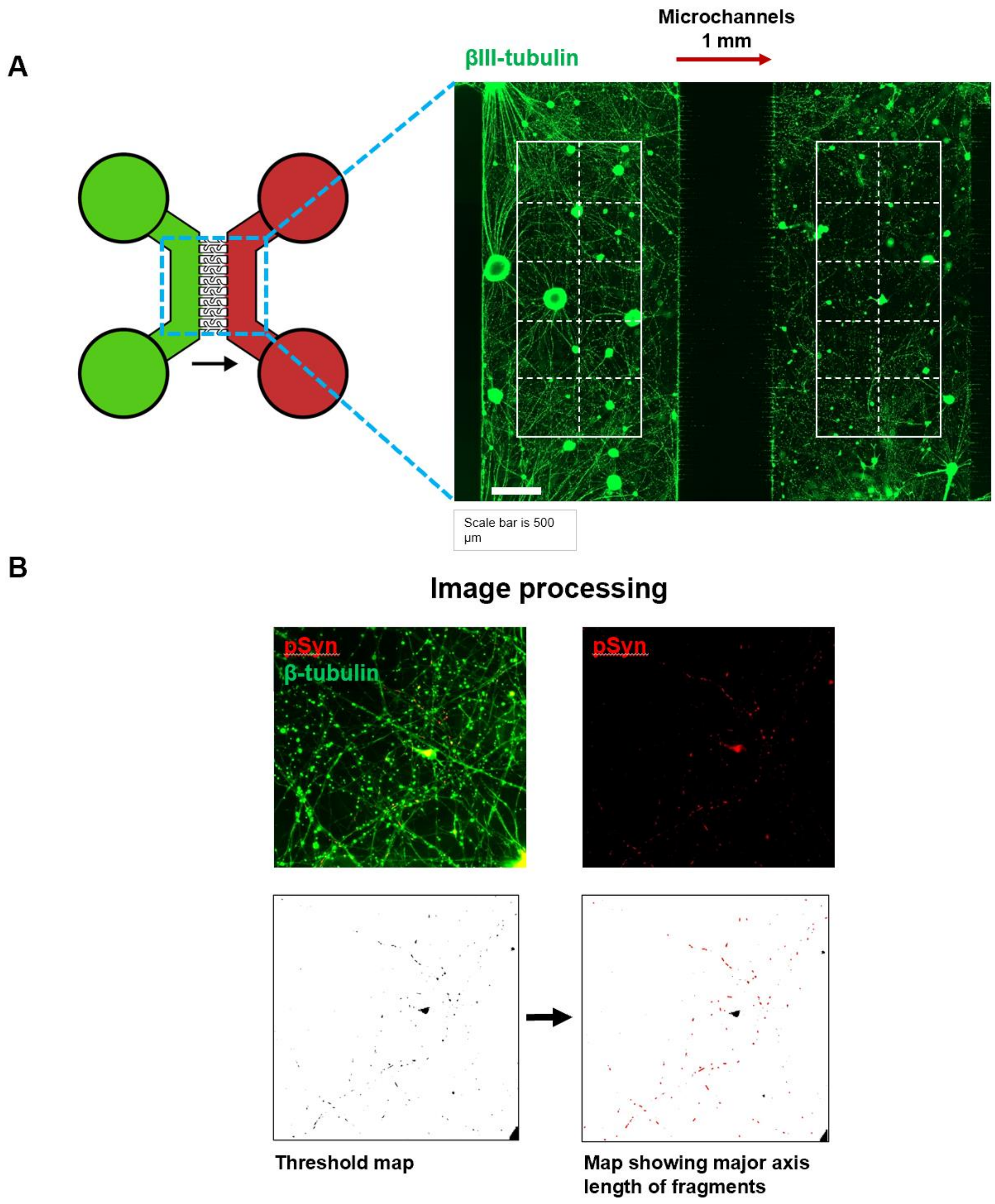

Supplementary figure 6 Analysis of microscopy images. A) Schematic showing the area of the chamber imaged for further analysis. An example of the images from one device is shown on the right. A total of 63 images are taken, of which 10 on each side are processed for further analysis. These images are indicated by white lines. B) Illustration of the steps taken by the software developed for this analysis. First the immunohistochemistry images from the pSyn staining are turned into a binary picture using an adjustable threshold (bottom left). From this, fragments of pathology (pSer129-positive fragments) are identified, and unspecific staining excluded according to a set of parameters such that included fragments were above a certain area threshold, had a low level of circularity and had an axis length above a set value. In an additional step, the fragments were overlayed with a threshold map of the βIII-tubulin staining and any fragments not overlapping (i.e., not located on a neurite) were excluded.

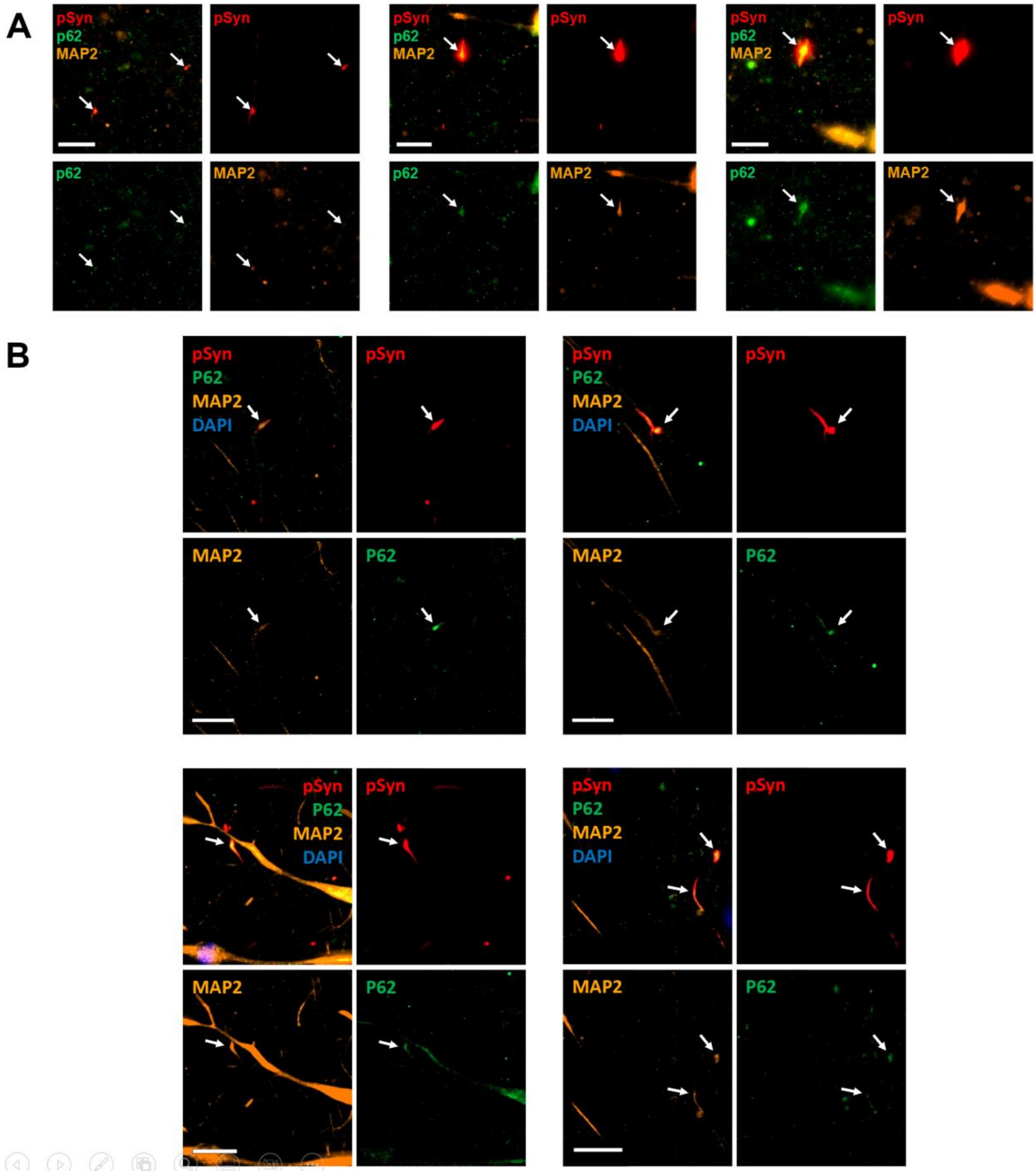

Supplementary figure 7 Colocalization of pSer129 pSyn, p62 and MAP2. A) Three examples of pathology showing increasingly strong signs of Lewy-body formation. pSyn staining is shown in red, MAP2 in orange and p62 in green. White arrows indicate colocalization of pSyn, MAP2 and p62. B) Additional apotome microscopy images to show colocalization of pSyn (red), P62 (green) and MAP2 (orange). Scale bar indicates 20 µm.

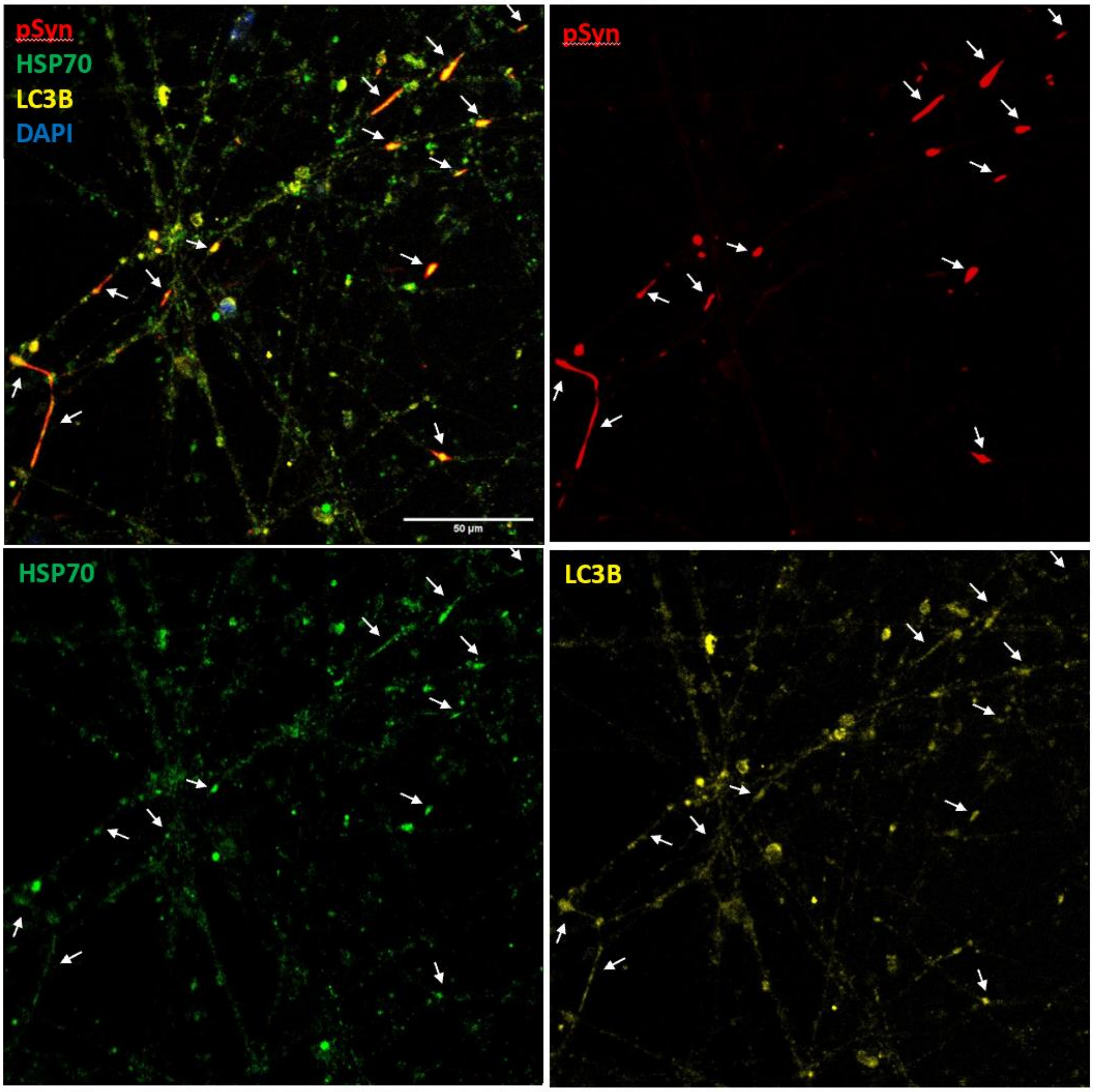

Supplementary figure 8 Confocal images showing colocalization of pSyn (red), HSP70 (green) and LC3B (yellow). Scale bar indicates 50 µm.

1% Triton-X; reverse (PFF-seeded) side

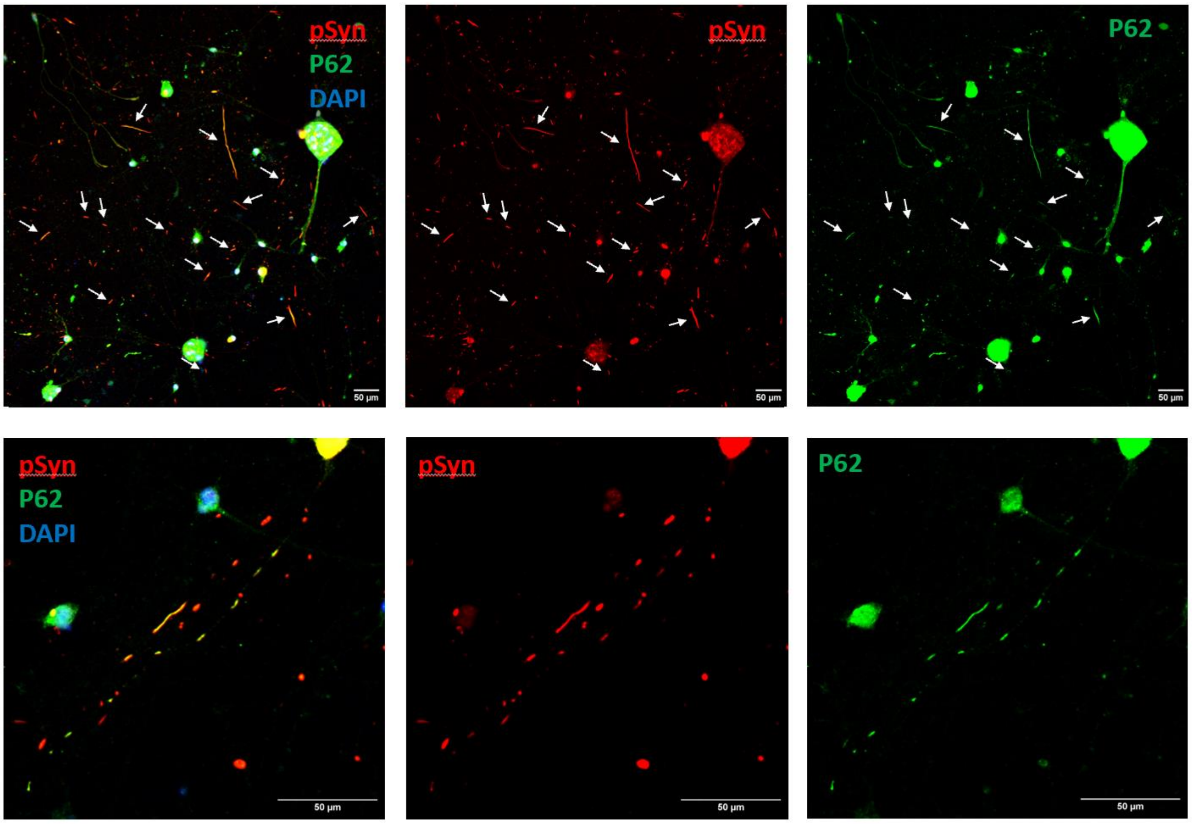

1% Triton-X; forward (non-seeded) side

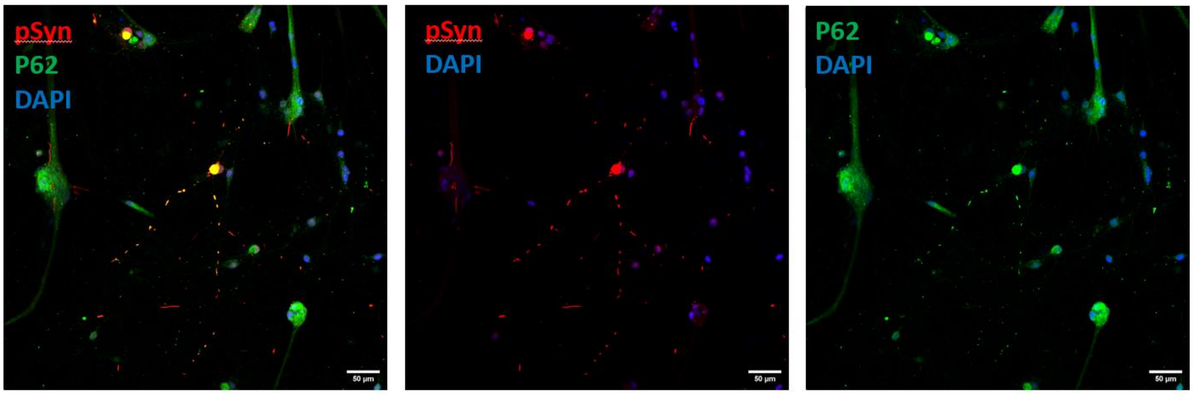

Supplementary figure 9 Confocal images showing detergent-resistant properties of pSyn pathology, both shown for the directly seeded reverse chamber and its neighbouring non-seeded forward chamber.

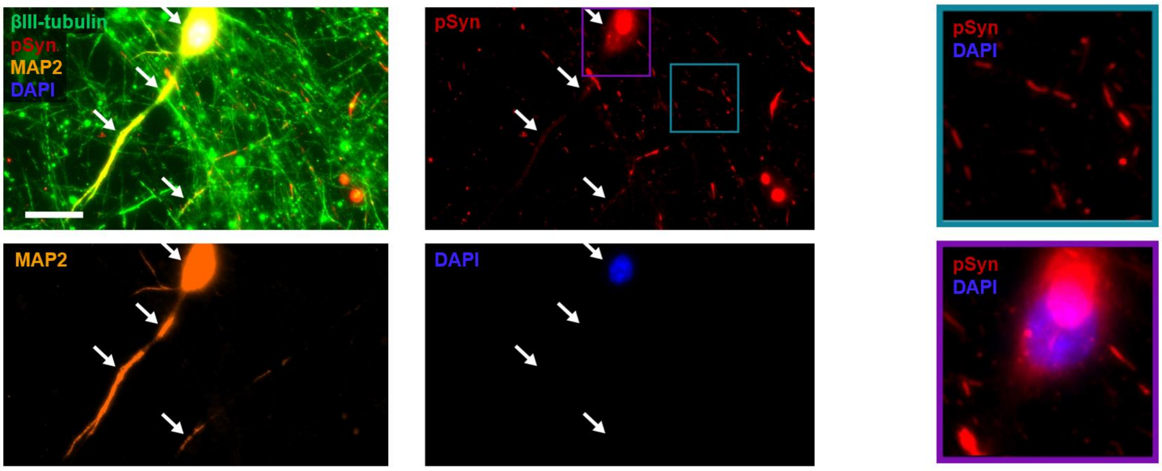

Supplementary figure 10 Characterization of pSyn pathology. βIII-tubulin staining is shown in green, pSyn in red, MAP2 in orange and nuclei were stained with DAPI in blue. The top white arrow indicates an example of unspecific pSyn staining of the soma. The other arrows indicate examples of unspecific staining on dendrites. The top inset (turquoise) shows examples of pSyn aggregates along neurites. In both the PFF-seeded and unseeded cultures, cell somas often showed weak background immunostaining for pSer-129-αSyn (purple square). This type of staining was excluded for quantitative analysis. The example also shows a bright inclusion that is likely to be an accumulation of pSer-129-positive αSyn. The scale bar is 20 µm.

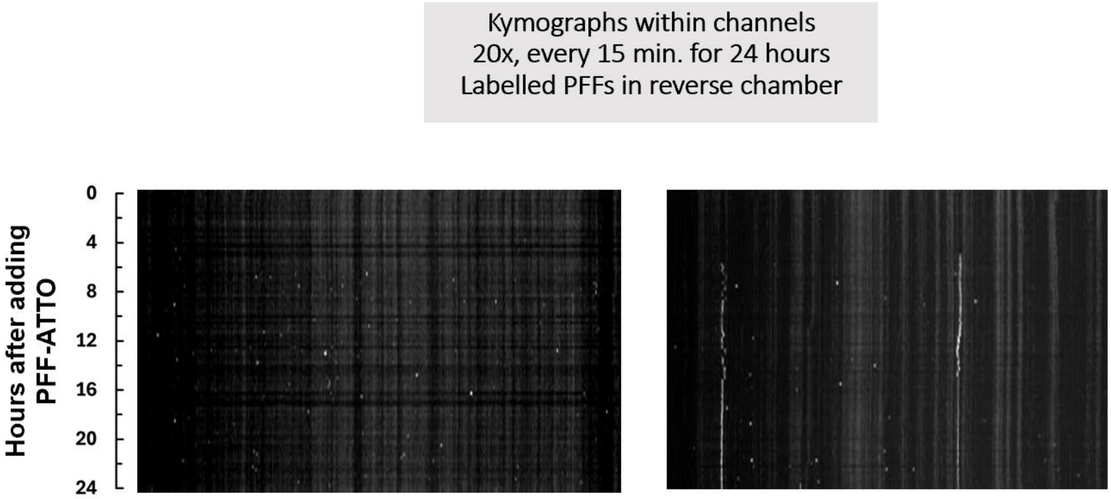

Supplementary figure 11 Kymographs within microchannels of a timelapse over 24 hours. Images were taken every 15 minutes. PFF-ATTO was added to the reverse chamber. 2 devices.

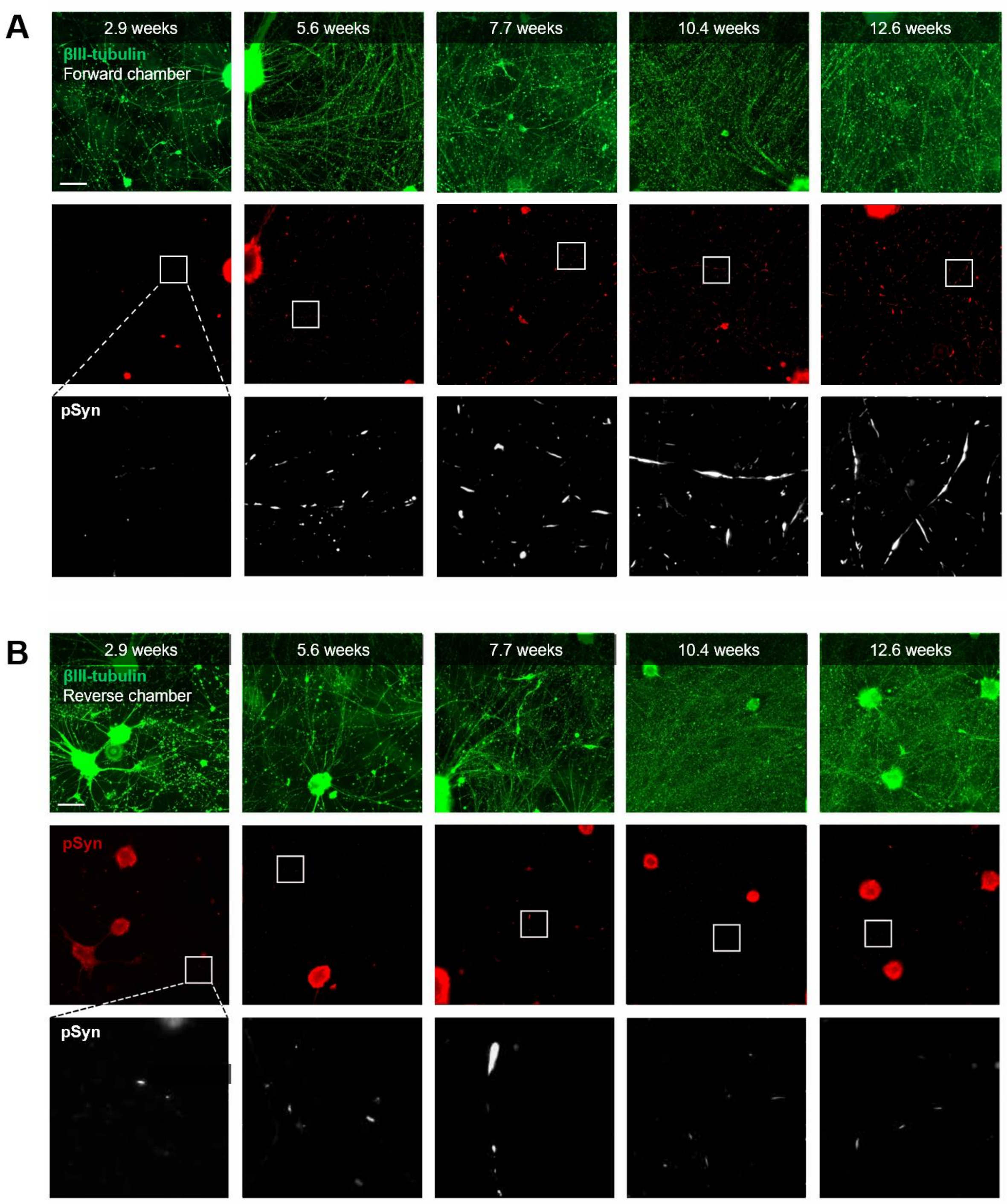

Supplementary figure 12 pSyn pathology increases with time in the directly seeded forward chamber, but an increase is hard to detect in the reverse chamber. A) Expansion of figure 5A, PFFs were added to the forward chamber. B) the same figure but then for the unseeded reverse chamber.

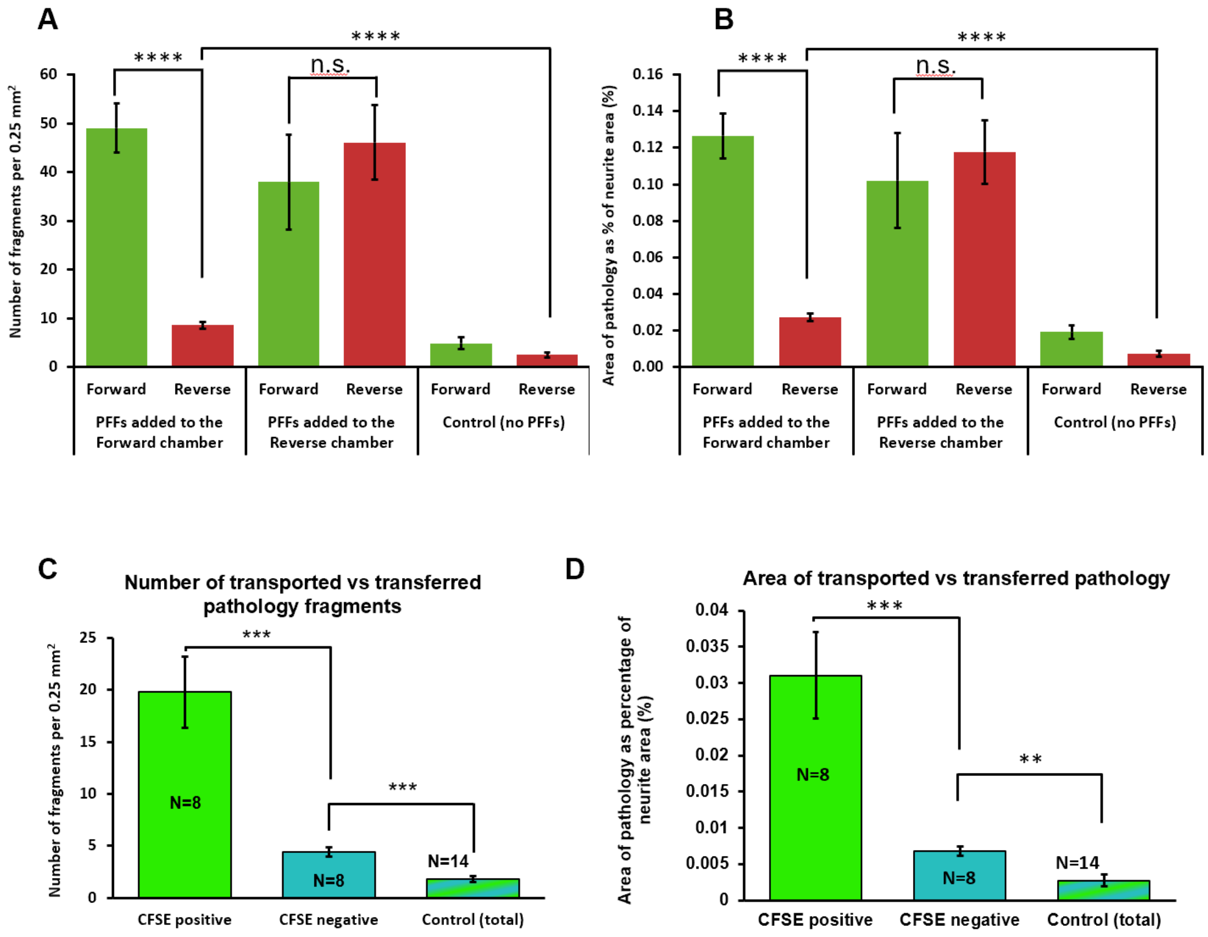

Supplementary figure 13 Different methods of quantification lead to similar results to the quantification as area of pathology per 0.25 mm^2. A&B) Figure 6C, quantifying respectively the number of fragments per 0.25 mm^2 and area of pathology as percentage of neurite area. PFFs added to forward chamber: n=34; PFFs added to the reverse chamber: n=25; Control: n=11. C&D) Figure 7B, quantifying respectively the number of fragments per 0.25 mm^2 and area of pathology as percentage of neurite area. PFFs added to the forward chamber: n=8; Control: n=14.

Statistics:

A) Non-seeded reverse vs. reverse control: mean difference=6.075, p= 8.418e-6. Non-seeded forward vs. forward control: mean difference=33.096, p= 0.000298. Non-seeded forward vs. seeded reverse: mean difference=8.092, p= 0.154.

B) Non-seeded reverse vs. reverse control: mean difference=0.020, p= 6.626e-6. Non-seeded forward vs. forward control: mean difference=0.083% of neurite area, p= 0.00224. Non-seeded forward vs. seeded reverse: mean difference=0.016%, p= 0.0775.

C) CFSE-positive vs. CFSE-negative: mean difference=15.735, p= 0.000771. CFSE-negative vs. Control: mean difference=2.606, p= 0.000721.

D) CFSE-positive vs. CFSE-negative: mean difference=0.0243%, p= 0.000155. CFSE-negative vs. Control: mean difference=0.004%, p= 0.00213.

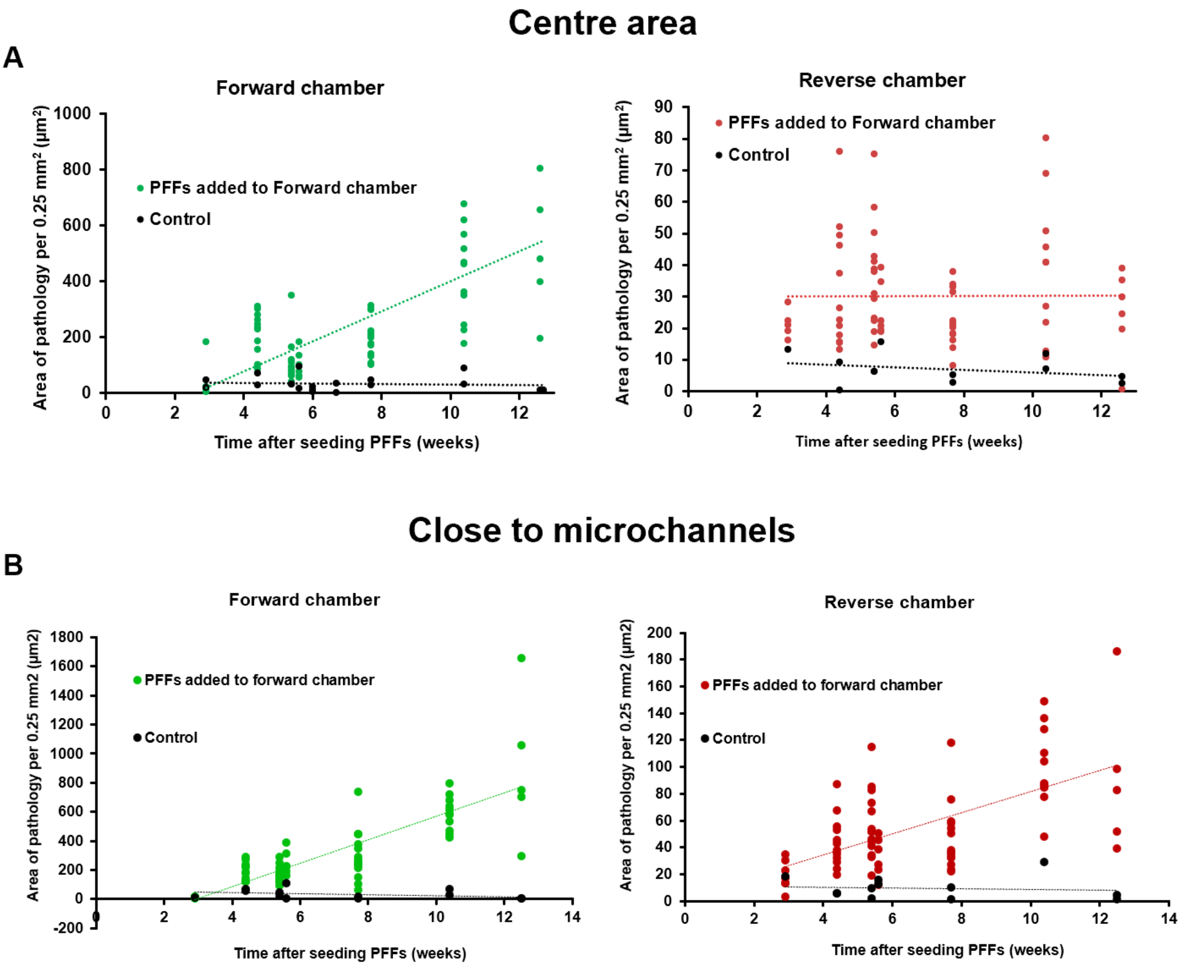

Supplementary figure 14 Pathology levels quantified after increasingly long incubation times after adding PFFs to the forward chamber. The forward chamber is shown on the left and the reverse chamber on the right. A) Data analysis was performed on images in the centre area of the chamber (an area between 400µm-1600µm away from the microchannels; forward: n=69, reverse: n=68). B) Analysis was conducted in an area between the microchannels and 400µm away (forward: n=69, reverse: n=68).

Statistics:

A, forward) PFFs: y= 53.828x - 137.706, F(1,67)= 68.460, p=7.715e-12, R^2=0.505; Control: y=-1.492x + 50.331, F(1,12)=0.401, p=0.539, R^2=0.032

A, reverse) PFFs: y=0.0224x + 30.051, F(1,66)=0.000978, p=0.975, R^2=0.00148, Control: y=24.531x - 1.733, F(1,16)=1.180, p=0.294, R^2=0.0687

B, forward) PFFs: y=80.718x -238.358, F(1,67) = 39.207, p= 1.110e-16, R^2= 0.642, Control: y=-3.464x + 60.175, F(1,11)=1.341, p=0.271, R^2=0. 109

B, reverse) PFFs: y=7.821x + 3.325, F(1,67) = 39.207, p= 3.097e-8, R^2= 0.369, Control: y=-0.260x + 11.500, F(1,10)=0.100, p=0. 758, R^2=0. 00994

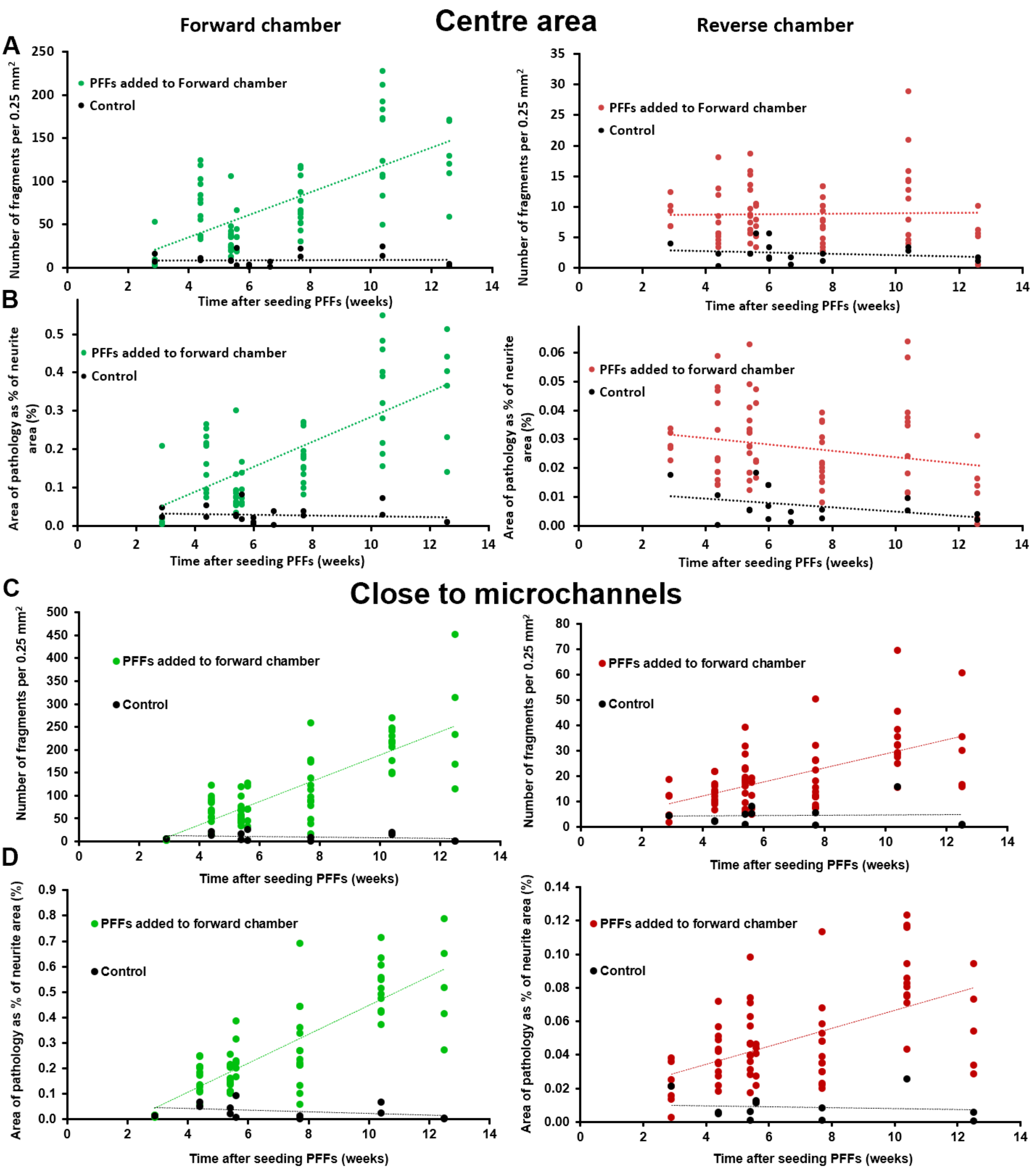

Supplementary figure 15 Same figure as Suppl. Fig. 11, but then quantifying the number of fragments per 0.25 mm^2 (A & C) and area of pathology as percentage of neurite area (B & D). (forward: n=69, reverse: n=68).

Statistics:

A, forward) PFFs: y=12.941x - 16.656, $F(1,67)=56.127$, $p=1.983e-10$, $R^2=0.456$; Control: y=-0.135x + 12.371, $F(1,12)=0.044$, $p=0.838$, $R^2=0.004$

A, reverse) PFFs: y=0.0327x +8.577, $F(1,66)=0.0228$, $p=0.881$, $R^2=0.000345$; Control: y=-0.106x + 3.162, $F(1,16)=0.613$, $p=0.445$, $R^2=0.0369$

B, forward) PFFs: y=0.0325x – 0.0417, $F(1,67)=64.003$, $p=2.405e-11$, $R^2=0.489$; Control: y=-0.000951x + 0.0340, $F(1,18)=0.269$, $p=0.611$, $R^2=0.0147$

B, reverse) PFFs: $y=-0.00109x+0.0347$, $F(1,66)=3.321$, $p=0.0730$, $R^2=0.0479$; Control: $y=-0.000771x+0.0126$, $F(1,16)=2.707$, $p=0.119$, $R^2=0.145$

C, forward) PFFs: $y=25.341x-65.146$, $F(1,67)=128.234$, $p<2.2e-16$, $R^2=0.657$; Control: $y=-0.728x+15.547$, $F(1,11)=0.857$, $p=0.374$, $R^2=0.0723$

C, reverse) PFFs: $y=-0.00109x+0.0347$, $F(1,66)=3.321$, $p=0.0730$, $R^2=0.0479$; Control: $y=-0.000771x+0.0126$, $F(1,16)=2.707$, $p=0.119$, $R^2=0.145$

D, forward) PFFs: $y=0.0568x-0.121$, $F(1,67)=136.565$, $p<2.2e-16$, $R^2=0.671$; Control: $y=-0.00302x+0.0538$, $F(1,11)=1.308$, $p=0.277$, $R^2=0.106$

D, reverse) PFFs: $y=0.00533x+0.0131$, $F(1,67)=27.298$, $p=1.862e-6$, $R^2=0.290$; Control: $y=-0.000303x+0.0126$, $F(1,10)=0.152$, $p=0.705$, $R^2=0.0150$

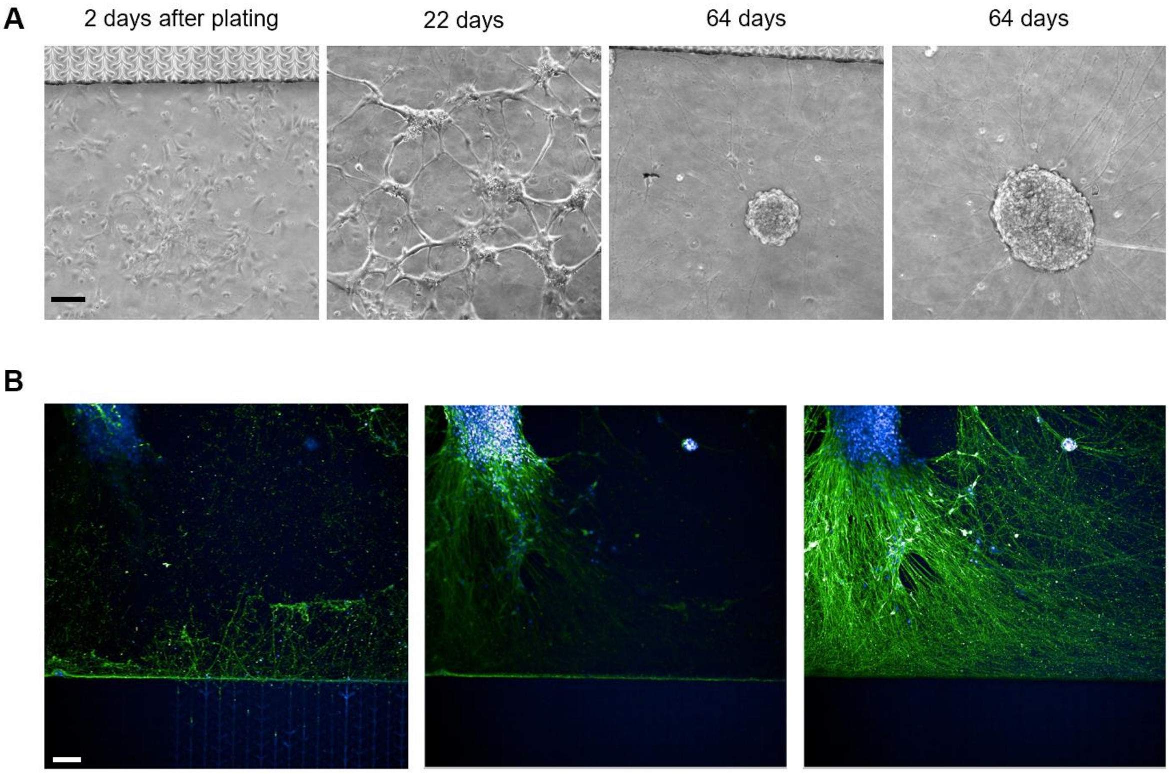

Supplementary figure 16 The growth and maturation of dopaminergic neurons in devices. A) Phase microscopy images of the culture in a device after 2 days of culturing, 22 days and 64 days. The third image shows an example of a small rosette comprised of neurons and the last image depicts a larger example of such a structure. The scale bar is 100 µm. B) Confocal microscopy image stack acquired from an immunostained device using an Opera Phenix (20x water-immersion objective). Blue is DAPI and green is βIII-tubulin. The inter-slice distance between image 1 and 2 is 40 µm and between image 2 and 3 30 µm. The scale bar is 100 µm.

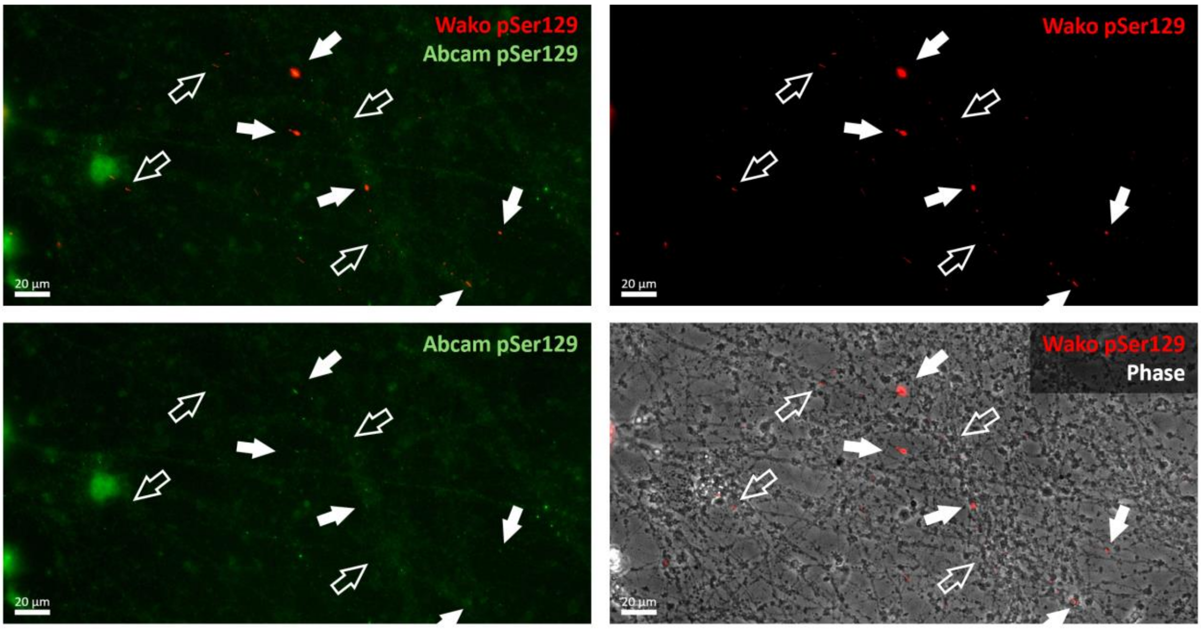

Supplementary figure 17 Immunohistochemical staining using the pSer129 antibody from Wako (red) and Abcam (ab51253, green). The filled arrows indicate colocalization of both antibodies and the open arrows indicate examples of Wako-positive fragments that have the characteristics of pathology (thread-like and following a neurite) that do not show colocalization.

Supplementary Videos 1A & 1B

Timelapse over 24 hours presented in Figure 1E showing mouse neurons expressing GFP cultured in the forward chamber and in the reverse chamber.

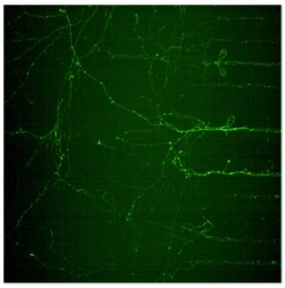
Video 1A: field of view showing forward-originating axons entering the reverse chamber, acquisition rate is every 15 min for 24 hours.

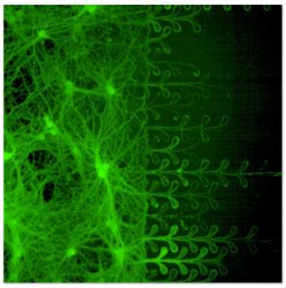
Video 1B: field of view showing reverse-originating axons becoming trapped inside the fine leaves pattern in the microchannels, acquisition rate is every 15 min for 24 hours.

Supplementary Videos 2

Timelapse presented in figure 2 for Brightfield and PFF-ATTO dye overlayed and just the PFF-ATTO dye.

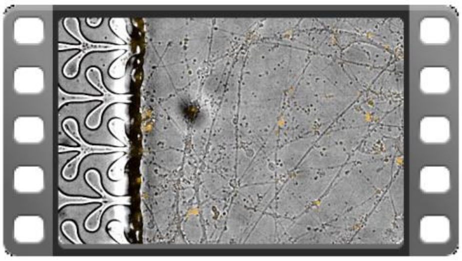
Video 2A: field of view showing labelled-PFF movement within a directly PFF-seeded chamber, acquisition rate is every 15 min for 24 hours. Overlay of brightfield and fluorescent channel.

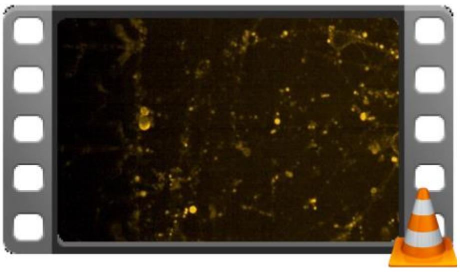
Video 2B: field of view showing labelled-PFF movement within a directly PFF-seeded chamber, acquisition rate is every 15 min for 24 hours. Fluorescent channel only as in Video 2A.
